# Engineering controllable alteration of malonyl-CoA levels to enhance polyketide production

**DOI:** 10.1038/s41589-025-01911-6

**Published:** 2025-06-11

**Authors:** Sarah H. Klass, Mia Wesselkamper, Aidan E. Cowan, Namil Lee, Nathan Lanclos, Seokjung Cheong, Zilong Wang, Yan Chen, Jennifer W. Gin, Christopher J. Petzold, Jay D. Keasling

**Affiliations:** 1 Joint BioEnergy Institute, Emeryville, CA USA; 2 Biological Systems and Engineering Division, Lawrence Berkeley National Laboratory, Berkeley, CA USA; 3 Department of Chemical & Biomolecular Engineering, University of California, Berkeley, Berkeley, CA USA; 4 Department of Bioengineering, University of California, Berkeley, Berkeley, CA USA; 5 Department of Molecular and Cell Biology, University of California, Berkeley, Berkeley, CA USA; 6 QB3, University of California, Berkeley, Berkeley, CA USA; 7 Centre for Biosustainability, Danish Technical University, Lyngby, Denmark

**Keywords:** Synthetic biology, Metabolic pathways, Biosynthesis

## Abstract

Heterologous expression of polyketide synthase (PKS) genes in *Escherichia coli* has enabled the production of various valuable natural and synthetic products. However, the limited availability of malonyl-CoA (M-CoA) in *E. coli* remains a substantial impediment to high-titer polyketide production. Here we address this limitation by disrupting the native M-CoA biosynthetic pathway and introducing an orthogonal pathway comprising a malonate transporter and M-CoA ligase, enabling efficient M-CoA biosynthesis under malonate supplementation. This approach substantially increases M-CoA levels, enhancing fatty acid and polyketide titers while reducing the promiscuous activity of PKSs toward undesired acyl-CoA substrates. Subsequent adaptive laboratory evolution of these strains provides insights into M-CoA regulation and identifies mutations that further boost M-CoA and polyketide production. This strategy improves *E. coli* as a host for polyketide biosynthesis and advances understanding of M-CoA metabolism in microbial systems.

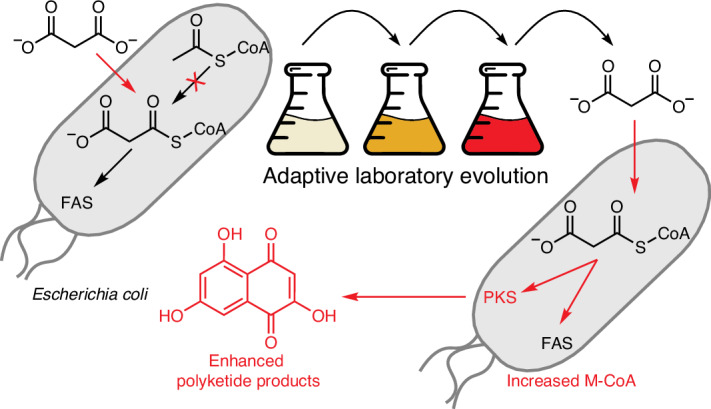

## Abstract (web summary)

Malonyl-CoA (M-CoA) is essential for polyketide biosynthesis, but its limited availability constrains production. Here the authors engineer and evolve an orthogonal M-CoA pathway in *Escherichia coli* to improve M-CoA metabolism, increasing M-CoA levels and polyketide yields.

## Main

The expression of native or hybrid PKSs in *Escherichia coli* offers a wide range of advantages and opportunities for diverse applications. In their natural hosts, PKSs produce valuable polyketide-derived natural products, including antibiotics, anticancer agents, immunosuppressants and other bioactive compounds^1^. Type III PKSs perform iterative decarboxylative Claisen condensation of acyl-CoA substrates^2^, whereas type I PKSs consist of multiple modular domains responsible for incorporating specific acyl-CoA derivatives into the growing polyketide chain^3^. Hybrid PKSs, combining modules or domains with distinct substrate specificities derived from various natural biosynthetic gene clusters^4^, can be rationally assembled to biosynthesize diverse and complex structures, accessing a broader chemical space than what is achievable by chemical synthesis alone^5–7^. Moreover, hybrid PKSs can be tailored to be compatible with specific microbial chassis, such as *E. coli*, enabling industrial-scale production in pharmaceuticals, agriculture and biomaterials, exemplified by the production of 6-deoxyerythronolide^8^, resveratrol^9^, naringenin chalcone^10^ and various lactones^7,11,12^.

Unlike microbial hosts that naturally produce PKSs, such as those from the *Streptomyces* genus^8,13^, *E. coli* is not a natural PKS host and, despite its versatility, faces a number of engineering challenges, including increased metabolic burden upon protein expression, lack of native phosphopantetheinyl transferases (PPTases) for PKS activation and limited acyl-CoA availability. Strategies to enhance PKS expression and activity include genetic code optimization^14^, improved hybrid PKS junctions^5^ and tuning transcription and translation by optimizing promoter strength^15^ and gene copy number^16,17^. However, even the most functional PKSs will fail to produce the desired product if the necessary CoA substrates are limited. In *E. coli*, one of the most commonly used PKS substrates, malonyl-CoA (M-CoA), is tightly regulated and primarily used by fatty acid (FA) biosynthesis, leading to a relatively small pool of free M-CoA and low production of M-CoA-based polyketides^18^.

Many strategies for enhancing the substrate pool of M-CoA have been established, as it has been identified as a clear bottleneck limiting the production of M-CoA-derived products such as polyketides and FAs^19^. Some methods have focused on increasing the availability of acetyl-CoA (Ac-CoA), a precursor for M-CoA, and/or overexpressing or supplementing enzymes responsible for converting Ac-CoA to M-CoA, such as the Ac-CoA carboxylase (ACC) complex^20–23^. Other genetic loci that are implicated in the diversion of carbon away from M-CoA have been targeted by CRISPRi to repress expression and successfully shift metabolic flux toward M-CoA synthesis^24^. Alternatively, exogenous supplementation of the potent FA synthase (FAS) inhibitor cerulenin is used to divert M-CoA toward polyketide production; however, cerulenin also covalently binds to the ketosynthase domains of PKSs in a similar manner to FAS, resulting in an inefficient solution to increase polyketide titers^25,26^. Finally, several methods to bypass the endogenous route to M-CoA via the ACC complex have been developed by expressing various malonate assimilation pathways, which have been shown to increase M-CoA levels and their subsequent incorporation into polyketides^27–30^.

Here, we engineer two PKS-compatible *E. coli* strains, K207-3 and BAP1, to enable a simple and controlled strategy of increasing M-CoA levels and various downstream PKS products. Both strains contain the PPTase gene required for PKS activation, *sfp*, from *Bacillus subtilis* and the native *prpE*, whose gene product converts propionate into propionyl-CoA, under an inducible T7 promoter. K207-3 also contains the genes encoding the propionyl-CoA carboxylase from *Streptomyces coelicolor*, enabling the conversion of propionyl-CoA to methylmalonyl-CoA (mM-CoA)^8,31^.

To provide exogenous control over M-CoA levels, we integrate genes for the widely used^30,32–35^ malonate assimilation pathway consisting of the malonate importer, *matC*, and the malonate:CoA-ligase, *matB*, into both genomes, resulting in a plasmid-free strain whose M-CoA and mM-CoA levels are directly tunable by exogenous addition of malonate and propionate, respectively. Furthermore, we achieve complete control over the M-CoA pool by inhibiting the endogenous M-CoA biosynthesis by removing *bioH*, whose gene product is required for biotin biosynthesis^36,37^ and, consequently, for activating the ACC complex that normally converts Ac-CoA to M-CoA and, thus, is necessary for cell growth^38^. By introducing the orthogonal MatBC pathway into this auxotrophic strain, the exogenous addition of malonate generates M-CoA and rescues cell growth. Similarly, malonate importers have been used in other biotin-independent *E. coli* strains to allow for the overexpression of the biotin-sequestering and, thus, toxic protein streptavidin^39^. Leveraging this malonate-dependent growth phenotype, we perform adaptive laboratory evolution (ALE), leading to evolved strains that accumulate mutations that increase M-CoA and PKS titers relative to the nonmutagenized strain. Among these are mutations previously identified using methods such as CRISPRi^24^, biosensor-guided random transposon libraries^40^ or metabolic modeling^23^ to improve M-CoA, FA or polyketide titers, in addition to several mutations in related pathways that have not yet been explored in M-CoA or PKS engineering strategies.

This work highlights the power of combining orthogonal control of metabolic pathways with ALE to enhance PKS precursor supply and polyketide titers while also deepening our understanding of M-CoA metabolism in microbial systems.

## Results

### Evaluation of malonate transporters in *E. coli*

Plasmids encoding the malonate transporters MadLM^41^, MdcF^42^ and MatC^35^ were examined for their ability to import malonate into *E. coli* and convert it to M-CoA by MatB. Production of M-CoA was quantified using the type III PKS 1,3,6,8-tetrahydroxynaphthalene (THN) synthase (RppA), which converts M-CoA to THN, spontaneously forming flaviolin. Flaviolin, a red compound, has distinct absorbance peaks at 340 nm and 520 nm and has served as an indirect indicator of intracellular M-CoA levels available for PKS biosynthesis^43,44^. The arabinose-inducible, highly active RppA variant plasmid (pBADT–RppA-NT)^44^ was cotransformed with each of the plasmids pCKmatBC, pBba2c–MatB–MadLM or pBbA2c–MatB–MdcF into BAP1, generating strains BAP1–pMatBC–RppA, BAP1–pMatB–MadLM–RppA and BAP1–pMatB–MdcF–RppA, respectively. Induction with anhydrotetracycline (aTc) caused growth toxicity in MadLM and MdcF, whereas no such toxicity was observed for MatC. These strains were cultured without aTc to mitigate toxicity, relying on leaky expression. Under these conditions, all transporters increased flaviolin production with higher malonate concentrations (Supplementary Fig. 1). MatC was selected for further experiments owing to its lower apparent toxicity.

### Engineering controllable levels of M-CoA

**Fig. 1:**
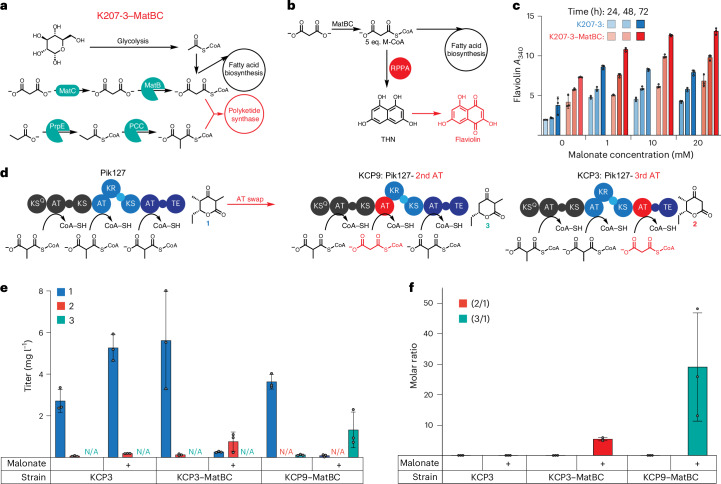
Schematic depiction of relevant pathways for MatBC-engineered *E. coli* strains. **a**, Integration of the MatBC pathway into K207-3 resulted in K207-3–MatBC, enabling modulation of M-CoA and mM-CoA pools by supplementing malonate and propionate, respectively. **b**, Pathway for flaviolin biosynthesis from M-CoA as a proxy for intracellular M-CoA levels. **c**, Absorbance measurements of the culture supernatant from three biological replicates at 340 nm (path length corrected to 0.1 cm^−^^1^). Maximum absorbance for K207-3–MatBC was observed at 72 h with 20 mM malonate supplementation (*A*_340_ = 13.09 ± 0.40), representing a 70% increase compared to K207-3 under the same conditions. **d**, Schematic depicting the predicted substrate specificities for Pik127 constructs with the acyltransferase (AT) domain from pikromycin module 3 exchanged at the second and third extension modules. **e**, Titers from three biological replicates of M-CoA-incorporated products 2 and 3 (*m*/*z* = 155) and the mM-CoA-incorporated product 1 (*m*/*z* = 169). For the Pik127-3rd acyltransferase construct, product 2 reached a maximum titer of 0.96 ± 0.12 mg l^−1^ in KCP3–MatBC supplemented with 20 mM malonate, a 14.8-fold relative to KCP3 without malonate (*P* = 0.0002). For the Pik127-2nd acyltransferase constructs, no product was detected in cultures of KCP9. However, in KCP9–MatBC cultures supplemented with 20 mM malonate, product 3 was observed with titers of 1.32 ± 0.85 mg l^−1^, 33-fold compared to the no-malonate condition, although the significant error prevented statistical significance (*P* = 0.0718). **f**, Molar ratios from three biological replicates of M-CoA-incorporated products (2 and 3) to the mM-CoA-incorporated product (1) were significantly increased in malonate-supplemented KCP3–MatBC and KCP9–MatBC cultures. Ratios of 2/1 (159-fold, *P* < 0.0001) and 3/1 (616-fold, *P* = 0.0475) were observed compared to no-malonate conditions. All data are presented as mean ± s.d. Statistical comparisons between groups were performed using GraphPad data analysis software with an unpaired *t*-test, assuming a Gaussian distribution, to calculate *P* values and determine statistical significance. Source data

The *matC* and *matB* genes from *Rhizobium trifolii* under the control of a *lacUV5* promoter were integrated via homologous recombination into the intergenic region downstream of *ompW*, a safe site for genomic integration and expression of recombinant proteins^45^, creating K207-3–MatBC. This genomic integration enabled tunable increases in M-CoA and mM-CoA levels by adding malonate and propionate to the medium, respectively (Fig. 1a). Similar to the plasmid-based system, K207-3 and K207-3–MatBC were transformed with pBADT–RppA-NT and monitored for their flaviolin production over 3 days in Lysogeny broth (LB) medium (10 µM isopropyl β-d-1-thiogalactopyranoside (IPTG), 0.2% arabinose and 0–20 mM malonate). K207-3–MatBC consistently produced more flaviolin than K207-3 across all malonate concentrations and a 70% increase (*P* = 0.0002) at 20 mM malonate. The increase in flaviolin titers correlated with malonate levels, demonstrating tunable enhancement of polyketide production (Fig. 1b).

### Production of polyketides derived from mM-CoA and M-CoA

PKSs use acyltransferase domains to select CoA substrates to be incorporated into the growing polyketide chain. Replacing native acyltransferase domains with those that use alternative acyl-CoAs can modify the final compound synthesized. However, previous attempts discussed in ref. ^46^ to replace the mM-CoA-specific acyltransferase domain of the first module (M1) of a shortened pikromycin synthase (Pik127)^47^ with an M-CoA-specific acyltransferase in a hybrid version resulted in the formation of both the mM-CoA and M-CoA-derived products, with the mM-CoA product being in excess. This was hypothesized to result from reduced acyltransferase activity or ketosynthase gatekeeping favoring mM-CoA elongation^46,48^. The full pikromycin PKS domain structure details are illustrated in Supplementary Fig. 2.

In addition to these factors, we hypothesized that the discrepancy in expected versus actual product might be due to an overabundance of mM-CoA during polyketide production in K207-3. To test this, *E. coli* strains K207-3 and K207-3–MatBC were transformed with plasmids encoding hybrid Pik127 variants with M-CoA-specific acyltransferase from pikromycin module 3 replacing native domains in module 2 (Pik127-2nd acyltransferase encoded on plasmids pBbS5k_pik12(pik_AT3) and pBbA5a_pik27) or module 3 (Pik127-3rd acyltransferase encoded on plasmids pBbS5k_pik12 and pBbA5a_pik27(pik_AT3)), resulting in the strains KCP9, KCP9–MatBC, KCP3 and KCP3–MatBC, respectively. Notably, the Pik127-3rd acyltransferase construct reinstalls the native ketosynthase–acyltransferase junction and should not suffer ketosynthase gatekeeping. Strains were cultured in EZ-rich medium with propionate (to generate mM-CoA) and ±malonate (to generate M-CoA). Liquid chromatography–mass spectrometry (LC–MS) analysis was performed to quantify the amount of 6-ethyl-3,5-dimethyldihydro-2H-pyran-2,4(3H)-dione (product 1), 6-ethyl-5-methyldihydro-2H-pyran-2,4(3H)-dione (product 2) and 6-ethyl-3-methyldihydro-2H-pyran-2,4(3H)-dione (product 3), and triacetic acid lactone (TAL) was used as an internal standard (Supplementary Figs. 3 and 4).

For the Pik127-3rd acyltransferase strains, supplementing malonate substantially increased the desired M-CoA-derived product (2) in KCP3–MatBC, reaching 0.96 ± 0.12 mg l^−1^, a 14.8-fold change relative to KCP3 without malonate (*P* = 0.0002). The ratio of product (2) to the mM-CoA product (1) was 5.3:1, representing a 150-fold change (*P* < 0.0001) over KCP3 (Fig. 1d,e).

For the Pik127-2nd acyltransferase strains (KCP9 and KCP9–MatBC), products 1 and 3 were only detected in KCP9–MatBC cultures. In this strain, malonate supplementation increased the M-CoA-derived product 3 by 33-fold compared to no supplementation, reaching 1.32 ± 0.85 mg l^−1^. The ratio of M-CoA product 3 to mM-CoA product 1 also improved significantly to 29:1, representing a 600-fold change relative to no supplementation (Fig. 1e). Exact quantification of product 3 was not possible owing to the lack of a specific chemical standard; instead, quantification relied on the chemically similar standard 6-ethyl-5-methyldihydro-2H-pyran-2,4(3H)-dione (Supplementary Fig. 4). The larger variability in product 3 titers may result from the reduced stability of the hybrid PKS, which contains multiple unnatural junctions, leading to inconsistent protein levels and product yields.

These results suggest that although acyltransferase folding and ketosynthase gatekeeping may influence substrate preference, increasing M-CoA availability via the MatBC pathway and malonate supplementation substantially enhance the incorporation of the intended substrate, M-CoA, into the final polyketide product.

### Engineering and recovery of M-CoA auxotrophy

**Fig. 2:**
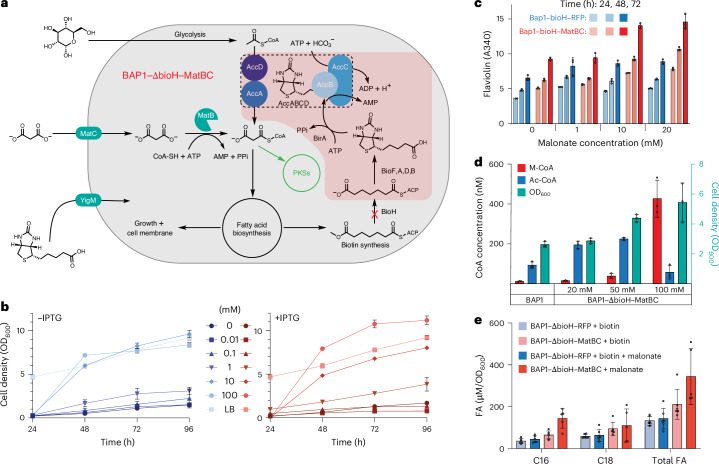
Increased polyketide production and growth rescue for malonate-supplemented MatBC strains. **a**, Integration of *matB* and *matC* at the *bioH* locus generated the biotin auxotrophic strain BAP1–ΔbioH–MatBC. In the absence of exogenous biotin, malonate becomes the sole precursor of M-CoA, as the glucose-derived Ac-CoA pathway is inactivated. **b**, Growth of three biological replicates of BAP1–ΔbioH–MatBC in induced (+10 µM IPTG) and uninduced conditions in M9 minimal medium (2% glucose) with increasing malonate concentrations (0–100 mM). Enhanced growth was observed at 1 mM malonate, whereas at ≥10 mM malonate, cell density after 48 h at 37 °C was comparable to LB-grown cultures. **c**, Flaviolin production from three biological replicates as a proxy for intracellular M-CoA levels. Absorbance at 340 nm (path length corrected to 0.1 cm^−1^) peaked at 72 h in BAP1–ΔbioH–MatBC supplemented with 20 mM malonate (*A*_340_ = 14.56 ± 1.06), representing a 60% increase compared to the control strain BAP1–ΔbioH–RFP under the same conditions. **d**, LC–MS quantification of M-CoA and Ac-CoA in BAP1 and BAP1–ΔbioH–MatBC from three biological replicates cultured in M9 minimal medium with increasing malonate concentrations for 36 h at 37 °C. M-CoA levels in BAP1–ΔbioH–MatBC were significantly higher than in BAP1 at 50 mM (3.3-fold, *P* = 0.016) and 100 mM malonate (36-fold, *P* = 0.0015). When normalized to DCW, M-CoA levels in BAP1–ΔbioH–MatBC were 2.0-fold (*P* = 0.078, not significant) and 18.1-fold (*P* = 0.0053) higher than in BAP1 at 50 mM and 100 mM malonate, respectively. **e**, FA titers from three biological replicates measured by GC–MS in BAP1–ΔbioH–RFP and BAP1–ΔbioH–MatBC cultured in M9 minimal medium supplemented with biotin and ±20 mM malonate. In malonate-supplemented cultures, BAP1–ΔbioH–MatBC exhibited significantly higher (*P* < 0.05) C16 and total FA levels than BAP1–ΔbioH–RFP. Total FA content included carbon chain lengths greater than 12, encompassing unsaturated and cyclopropanated isoforms. Statistical comparisons between groups were performed using GraphPad data analysis software with an unpaired *t*-test, assuming a Gaussian distribution, to calculate *P* values and determine statistical significance. Source data

To precisely control M-CoA levels in *E. coli*, the endogenous M-CoA biosynthesis pathway, mediated by the biotin-activated ACC complex, was inhibited and replaced with the MatBC pathway. The *matB* and *matC* genes were integrated into the BAP1 genome, replacing the essential biotin pathway gene *bioH*, to create the strain BAP1–ΔbioH–MatBC. This auxotrophy was rescued either by adding biotin (present in media like LB and transported intracellularly via YigM^49^) or by supplementing malonate, which is imported and converted to M-CoA by the MatBC pathway (Fig. 2a).

Growth rescue by malonate was tested in BAP1–ΔbioH–MatBC using M9 minimal medium with 2% glucose and increasing malonate concentrations (up to 100 mM). The effect of IPTG induction of the *lacUV5* promoter on *matBC* expression was also evaluated. Substantial growth was observed with as little as 1 mM malonate, both with and without IPTG induction. At concentrations of ≥10 mM malonate, cell density comparable to LB-grown controls was achieved after 48 h at 37 °C, despite slower growth at high malonate levels during the first 24 h (Fig. 2b).

Flaviolin production, used as a proxy for intracellular M-CoA levels, was measured in BAP1–ΔbioH–MatBC and a control strain, BAP1–ΔbioH–RFP (where *bioH* was replaced with the red fluorescent protein gene). Both strains, transformed with pBADT–rppA-NT, were cultured in LB (+10 µM IPTG and 0.2% arabinose) with increasing malonate concentrations for 3 days. Flaviolin production increased with malonate supplementation only in BAP1–ΔbioH–MatBC. At 20 mM malonate, BAP1–ΔbioH–MatBC produced 64% more flaviolin than BAP1–ΔbioH–RFP (*P* = 0.015; Fig. 2c).

### Quantification of acyl-CoAs and FAs

To quantify M-CoA levels directly, in the absence of a PKS, BAP1 and BAP1–ΔbioH–MatBC were cultured in M9 minimal medium with 2% glucose and increasing malonate concentrations for 36 h at 37 °C, reaching early stationary phase when M-CoA levels are typically low^50^. In BAP1–ΔbioH–MatBC, both the optical density of a sample measured at a wavelength of 600 nm (OD_600_) and M-CoA levels, measured via LC–MS, increased with malonate supplementation. The highest M-CoA concentration, 436.9 ± 57.5 nM, was observed with 100 mM malonate. Normalized to cell density, this corresponded to 0.206 ± 0.025 nmol mg^−1^ dry cell weight (DCW), an 18.1-fold change compared to 0.011 ± 0.09 nmol mg^−1^ DCW in BAP1 (Fig. 2d).

The impact of increased M-CoA levels on FA biosynthesis, FA profiles of BAP1–ΔbioH–MatBC and the control strain BAP1–ΔbioH–RFP were analyzed using gas chromatography (GC)–MS after 24 h of growth in M9 minimal medium at 37 °C with or without malonate supplementation. BAP1–ΔbioH–MatBC nearly depleted all exogenous malonate during the experiment, whereas substantial amounts remained in the medium of BAP1–ΔbioH–RFP (Supplementary Fig. 7). With malonate supplementation, BAP1–ΔbioH–MatBC produced substantially higher levels of C16 FAs and had a greater total FA content, including unsaturated and cyclopropanated FAs, compared to BAP1–ΔbioH–RFP (Fig. 2e).

### Production of uniformly labeled ^13^C polyketides

**Fig. 3:**
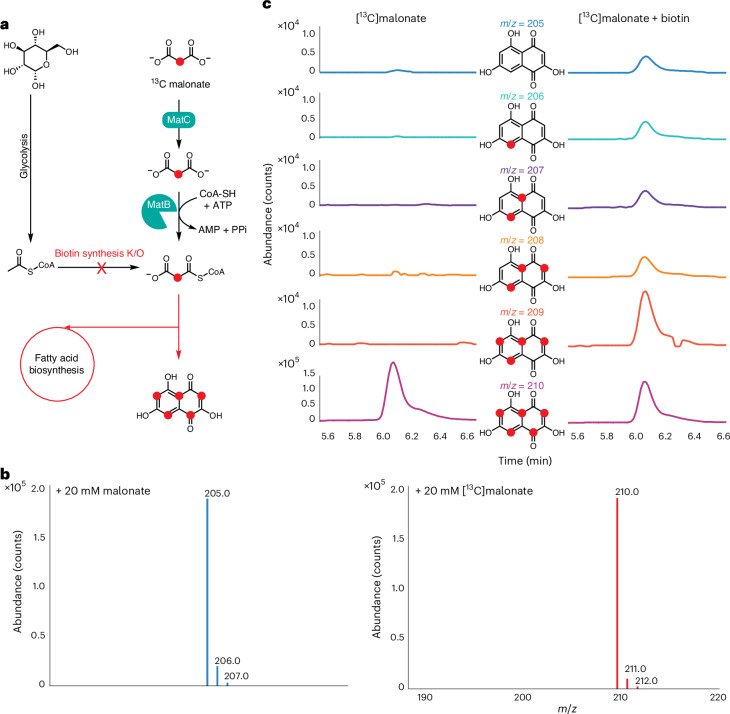
Production of uniformly labeled ^13^C-labeled PKS products using BAP1–ΔbioH–MatBC. **a**, Schematic representation of uniform isotopic labeling of M-CoA-derived polyketides. **b**, Extracted mass spectra of flaviolin synthesized in BAP1–ΔbioH–MatBC supplemented with unlabeled or ^13^C-labeled malonate. The incorporation of [^13^C]malonate results in a 5 Da shift, indicating uniform integration of five ^13^C-labeled M-CoA molecules. **c**, Comparative analysis of ^13^C-labeled isotopic incorporation in flaviolin produced by BAP1–ΔbioH–MatBC, with or without restoration of biotin auxotrophy through the exogenous addition of 25 mg l^−1^ biotin. After 24 h of cultivation in autoinduction M9 minimal medium at 37 °C, the strains achieved up to 99.8% purity for five-carbon ^13^C-labeled flaviolin (*m*/*z* = 210) in the absence of biotin. By contrast, biotin supplementation reduced the labeling efficiency to 79.9%, with additional peaks corresponding to mono (*m*/*z* = 206), di (*m*/*z* = 207), tri (*m*/*z* = 208) and tetra (*m*/*z* = 209) incorporation of ^13^C-labeled M-CoA. The 340 nm and 600 nm absorbances in a 1 cm light path were recorded at the final 72-h time point. For cultures supplemented with [^13^C]malonate, OD_600_ = 5.5, *A*_340_ = 14.78 and *A*_340_/OD_600_ = 2.7. In cultures supplemented with both [^13^C]malonate and biotin, OD_600_ = 5.1, *A*_340_ = 13.65 and *A*_340_/OD_600_ = 2.7. Source data

In BAP1–ΔbioH–MatBC, the engineered MatBC pathway is the sole means for generating M-CoA. This design facilitates the production of uniform isotopically labeled polyketides, free from contamination by unlabeled endogenously produced M-CoA without the need to cultivate in [^13^C]glucose (Fig. 3a). Here the flaviolin-producing strain BAP1–ΔbioH–MatBC–RppA was cultivated in M9 minimal medium supplemented with 2% unlabeled glucose with either unlabeled malonate or [^13^C]malonate labeled solely at position 2. A control without supplemental malonate failed to grow and produce flaviolin, while the strains supplemented with malonate turned turbid and red (Supplementary Fig. 5). LC–MS analysis of the cultures revealed a consistent 5-Da mass shift in the peak corresponding to flaviolin when [^13^C]malonate was used compared to the unlabeled counterpart, in accordance with the incorporation of five M-CoA molecules (Fig. 3b).

To demonstrate the necessity of preventing endogenous M-CoA biosynthesis for achieving uniform ^13^C labeling, biotin was introduced into the medium along with [^13^C]malonate to enable endogenous M-CoA production. Extracted ion chromatograms (EICs) for masses corresponding to flaviolin labeled with zero to five [^13^C]carbons were examined for cultures fed solely with [^13^C]malonate and those also supplemented with biotin. In the case of [^13^C]malonate alone, a prominent peak at 210 Da was observed, representing five [^13^C]M-CoA molecules with 99.8% purity over other isotopically labeled species. Conversely, when biotin was included in the medium, detectable peaks for masses corresponding to flaviolin with zero to five [^13^C]carbons were evident (Fig. 3c). EICs for masses 205–210 Da were similarly analyzed for controls where only malonate or biotin was added to the culture. In both cases, a predominant peak at 205 Da was observed, accompanied by minor peaks at 206 and 207, which is in line with the natural isotopic abundance of carbon (Supplementary Fig. 6). Over the 3-day period, the final OD_600_ for both strains exceeded 5.0, while the flaviolin absorbance at 340 nm exceeded 13.5, similar to what was observed in LB medium (Fig. 2b). Notably, when supplementing with both [^13^C]malonate and biotin over a 72-h period, the ratio of the uniformly labeled 210 Da peak progressively rose from 79.9% on day 1 to 92.6% by day 3 (Supplementary Fig. 6). This suggests that, over time, the M-CoA pool derived from supplemented malonate dominated the M-CoA derived from Ac-CoA.

### ALE of BAP1–ΔbioH–MatBC

ALE was performed on BAP1–ΔbioH–MatBC to enhance M-CoA production and availability by exploiting its growth dependency on exogenous malonate. The BAP1–ΔbioH–MatBC strain was transformed with the mutagenesis plasmid-6 (MP6), which harbors *dnaQ926*, *dam*, *seqA*, *emrR*, *ugi* and *cda1* and enables a 322,000-fold increase in the mutation rate of *E. coli* chromosomal DNA^51^. The strain was cultured in M9 minimal medium with 2% glucose and 1 mM malonate, the lowest concentration exhibiting growth recovery, in a Chi.Bio turbidostat^52^ where the culture’s OD_600_ was maintained at 0.75. After 7 days, the evolved culture was plated on M9 minimal agar plates with 3 mM malonate, and six strains (E1_S1–6) were further cultured and sequenced. Increased growth was observed for four of six strains, indicating positive growth adaptations (Supplementary Fig. 8).

In this first round of ALE, many mutations were observed in all strains relative to the nonmutagenized control. A common mutation in all strains was a single point mutation (C>A) to the lac operator upstream of the *matB* and *matC* genes, which is known to disrupt repressor affinity^53^. PCR amplification and sequencing of the total evolved pool revealed that 99.6% of the sequencing reads contained this lac operator mutation. This result, supported by proteomics data (Supplementary Data 1), indicates that increased expression of MatB and MatC provides a growth advantage in this context.

**Fig. 4:**
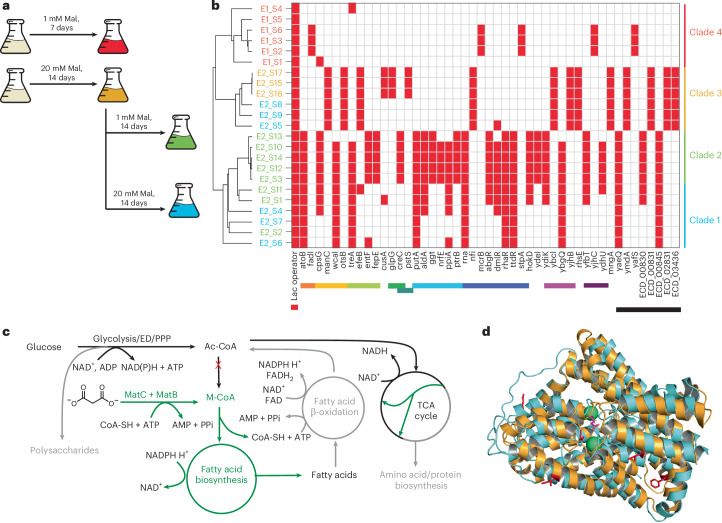
Mutations acquired through ALE. **a**, Schematic representation of the adaptive evolution conditions for 23 evolved strains (E1_S1–S6 and E2_S1–S17). **b**, Clustering of evolved strains based on 1,518 observed mutations. A dendrogram was constructed using Pearson’s correlation and an average linkage method to group strains into four distinct clades. The presence–absence matrix highlights representative frameshift or stop codon mutations occurring in more than 50% of strains within each clade. Red indicates the presence of a mutation, whereas white indicates its absence. Mutations are functionally categorized where possible with boxes of the corresponding colors next to the gene names—MatBC pathway (red), FA β-oxidation (orange), colanic acid/polysaccharide biosynthesis (gold), iron transport and metabolism (lime green), Membrane association (green), phosphate transport (blue–green), amino acid/protein biosynthesis (light blue), DNase/RNase/transcription regulation (dark blue), putative membrane association (magenta), alternative carbon sources (purple) and unknown function (black). **c**, Metabolic pathway analysis of ALE-derived mutations mapped onto central metabolism. Pathways with increased flux are indicated in green, and those with decreased flux due to frameshift or stop codon mutations are marked in gray. **d**, AlphaFold-predicted structure of MatC (gold), highlighting six missense mutations (A365S, F400S, V87A, I91N, L424P and P372L) observed in six separate strains. These mutations, identified as representative of cluster 1 by PCA analysis (Supplementary Fig. 9), are shown in red. The MatC structure is overlaid on the VcINDY transporter (Protein Data Bank (PDB) ID: 6OL1; cyan), with bound sodium (green) and succinate (pink) shown for reference. Mal, malonate.

Building on these findings, we extended ALE to explore additional conditions and a longer time frame. To prevent evolutionary escape and enable prolonged selection, a kanamycin-resistance marker was integrated into the genome, replacing additional biotin biosynthesis genes (*bioA*, *bioB*, *bioC*, *bioD* and *bioF*), creating BAP1–ΔbioH–MatBC–Δbio-kan. This strain was transformed with the MP6 plasmid and evolved for 2 weeks in M9 medium with 2% glucose and 20 mM malonate, a concentration that supports full growth recovery and high polyketide titers (Fig. 2c). After 2 weeks, the culture was split into the following two conditions: low malonate (1 mM) and high malonate (20 mM), for an additional 2 weeks. The experimental workflow is illustrated in Fig. 4a. Serial dilutions in test tubes were performed throughout the 4-week evolution. Individual colonies from each condition (E2_S1–17) were isolated and sent for whole-genome sequencing.

As in the previous ALE experiment, numerous mutations were identified in all evolved strains compared to the nonmutagenized control (Supplementary Data 2). All strains again carried a mutation in the *lac* operator upstream of *matBC*. However, in the 4-week evolution experiment, a substantially higher number of mutations were observed, with over 1,518 missense, frameshift or stop codon mutations identified across all 23 strains (E1_S1–6 and E2_S1–17). These mutations were transformed into an absence–presence matrix and analyzed using hierarchical clustering (Pearson correlation and average linkage method), resulting in a dendrogram with four distinct clusters (Fig. 4b, clades 1–4).

Strains evolved for 1 week in 1 mM malonate (E1_S1–S7) formed clade 4, which exhibited the fewest average mutations (41.33 per strain). In contrast, clade 2 (E2_S3, S10, S12, S13 and S14) contained strains that evolved in 20 mM malonate for 2 weeks, followed by 1 mM malonate for an additional 2 weeks, and had the highest average mutations (303 per strain). Clade 1 (E2_S1, S2, S4, S6, S7 and S11) included strains from both 1 mM and 20 mM malonate conditions during the second 2-week evolution. Clade 3 (E2_S5, S8, S9, S15, S16 and S17) contained strains that evolved exclusively in 20 mM malonate for 2 or 4 weeks before the split into two conditions.

Given the large number of missense mutations and limited functional characterization, our analysis focused on frameshift and stop codon mutations that resulted in the premature termination of protein translation. Mutations present in at least 50% of strains within a clade were considered representative and used to construct a presence–absence mutation matrix (Fig. 4b), with functional annotations assigned where possible.

Some of the observed mutations have been previously identified to improve free FA or M-CoA levels by their deletion or downregulation in *E. coli*, such as *atoB* (clades 1 and 2), *creC* (clade 2) and *glpG* (clade 3)^54^. The increased free FA titers were proposed to occur through reduction of flux through β-oxidation (*atoB*), downregulation of phosphate starvation response (*creC*) and downregulation of membrane proteins (*glpG* and *creC*), which might be associated with tolerance to free FAs^54–56^.

Additional mutations were observed in pathways previously linked to improved FA or M-CoA titers. Knockouts of iron transport genes, *fhuA* and *tonB*, have been shown to increase intracellular M-CoA levels and polyketide production, whereas iron supplementation reduces these levels^40^. In our study, frameshift or stop codon mutations were observed in genes involved in iron transport and metabolism, such as *efeB* (clades 3 and 1), *entF* (clades 2 and 1) and *fepE* (clade 2).

Similarly, *rcsA* knockouts, which redirect flux toward glycolysis by repressing colanic acid biosynthesis, have been reported to enhance M-CoA and polyketide titers^40^. Correspondingly, our strains harbored mutations associated with colanic acid biosynthesis genes *cpsG* (clades 1 and 2), *manC* (clade 3) and *wcaI* (clades 1 and 2).

Clade 4 also possessed a mutation to *fadI*, encoding a thiolase enzyme involved in β-oxidation. The specific role of *fadI* in β-oxidation remains debated, with evidence suggesting it functions on long-chain FAs under aerobic conditions or short-chain FAs anaerobically^57,58^. However, *fadI* knockouts do not completely disable β-oxidation activity, as both *fadI* and its homolog *fadA* must be inactivated to fully block FA catabolism^59,60^.

Mutations in genes related to amino acid metabolism, protein biosynthesis and nitrogen regulation, which could redirect carbon flux from protein synthesis to lipid production, were observed in all clades, with clades 1 and 2 being particularly enriched. Prior studies have shown that repressing ribosomal protein L23 (*rplW*) or tRNA-associated genes (*tyrU* and *rnpB*) increases FA production^54^. In our strains, frameshift or stop codon mutations in amino acid metabolism genes, such as *aldA* (lactaldehyde dehydrogenase, involved in the methylglyoxal pathway) and *putA* (proline dehydrogenase), were identified. Mutations in protein biosynthesis genes, including *ppiA* (peptidyl-prolyl isomerase A), *ptrB* (oligopeptidase B) and *rna* (RNaseI), were also observed. Additionally, a mutation in *nrfE*, a component of the nitrite reductase complex involved in nitrogen regulation, was detected.

A comparison with the mutational profiles of a previously reported ALE of *E. coli* in glucose minimal medium^61,62^ revealed no overlap of representative frameshift or stop codon mutations with our study. However, missense mutations in RNA polymerase genes, such as *rpoB* (E546V and E672K), *rpoC*^62^, *rpoD* (S253P) and *rpoA* (G279V)^61^, have been reported to alter promoter binding and broadly impact transcriptional levels, providing fitness advantages. In our dataset, missense mutations were found in *rpoB* (D853N, *n* = 11; K203R, *n* = 7, co-occurring with D853N), *rpoC* (L1059P, *n* = 1; S655Y, *n* = 1) and *rpoD* (A375T, *n* = 1). These findings align with previous observations of common mutations in the *rpoBC* operon during ALE^63,64^, suggesting adaptation to minimal glucose media alongside selective pressures such as M-CoA and FA limitations.

The evolved strains also carried numerous other missense mutations with unknown functional impacts. Of particular interest were six unique missense mutations in the MatC malonate importer (A365S, F400S, V87A, I91N, L424P and P372L) found in six strains (E2_S2, S3, S4, S6, S7 and S16). To visualize the potential locations of these mutations on the MatC protein, AlphaFold 2.3 was used to model the MatC structure, clustering 640 predicted structures into six representative cluster control models (Supplementary Fig. 9). The structure representing cluster 1 closely resembled the crystal structure of the sodium-dependent dicarboxylate transporter (*Vibrio cholerae* INDY (*Vc*INDY)) from *Vibrio cholerae*, a homodimer that undergoes structural rearrangement upon substrate binding^65^. Some mutations, such as P372L, were located on the protein exterior or in flexible regions (V87A and I91N). Others, like F400S (strain E2_S3), were near the Na^+^ substrate-binding site, whereas A365S and P372L aligned with the *Vc*INDY α-helical region involved in substrate binding and conformational changes (Fig. 4d). Further structural studies are needed to understand how these mutations affect the function and structure of MatC.

**Fig. 5:**
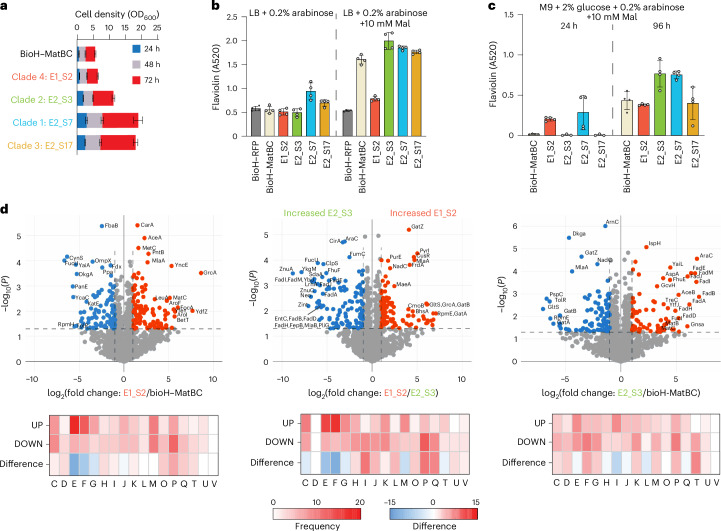
Characterization of evolved strains. **a**, Cell density measurements over 3 days for four biological replicates of representative strains from each clade of the dendrogram in Fig. 4. **b**,**c**, Flaviolin production from four biological replicates of representative strains from each clade in LB, LB + 10 mM Mal (**b**) and M9 + 10 mM Mal at 24 h and 96 h time points (**c**). Significantly increased (*P* < 0.05) titers compared to the unevolved bioH–MatBC strain were observed for E1_S2 in M9 + 10 mM Mal at 24 h; E2_S3 in LB + 10 mM Mal and M9 + 10 mM Mal at 96 h; E2_S7 in all conditions; and E2_S17 in LB, LB + 10 mM Mal and M9 + 10 mM Mal at 96 h. All data are presented as mean values ± s.d. Statistical comparisons between groups were performed using GraphPad data analysis software with an unpaired *t*-test, assuming a Gaussian distribution, to calculate *P* values and determine statistical significance. **d**, Proteomic analysis from four biological replicates comparing evolved and unevolved strains, showing COG assignments for proteins with significantly increased (fold change > 1, *P* < 0.05) or decreased (fold change < −1, *P* < 0.05) abundance, determined using Welch’s *t*-test correction. The number of proteins in each COG category is shown for UP and DOWN proteins, along with the difference between these counts (DOWN − UP = difference). COG functional categories—C, energy production and conversion; D, cell cycle control, cell division and chromosome partitioning; E, amino acid transport and metabolism; F, nucleotide transport and metabolism; G, carbohydrate transport and metabolism; H, coenzyme transport and metabolism; I, lipid transport and metabolism; J, translation, ribosomal structure and biogenesis; K, transcription; L, replication, recombination and repair; M, cell wall/membrane/envelope biogenesis; N, cell motility; O, post-translational modification, protein turnover and chaperones; P, inorganic ion transport and metabolism; Q, secondary metabolite biosynthesis, transport and catabolism; T, signal transduction mechanisms; U, intracellular trafficking, secretion and vesicular transport; V, defense mechanisms. UP, upregulated; DOWN, downregulated. Source data

Based on the genome sequencing and proteomics data (Fig. 5d), we propose the following hypothesis for the observed mutations (Fig. 4c): mutations in the lac operator increase *matB* and *matC* expression, enhancing flux toward M-CoA to support FA biosynthesis. Missense mutations to *matC* may further enhance this flux, although biocatalytic studies are needed for confirmation. Reduced glucose diversion to polysaccharide synthesis increases glucose availability for NADPH and ATP production. Decreased protein translation and biosynthesis (amino acids and RNA) align with the proteomic data of our evolved strains (Fig. 5d), indicating a shift toward the Entner–Doudoroff pathway, which uses fewer enzymatic steps than glycolysis and produces NADPH and NADH.

Mutations in iron transport genes increase flux through the glyoxylate shunt pathway enzymes (AceA and AceB) to bypass iron-dependent enzymes in the tricarboxylic acid (TCA) cycle. Mutations to β-oxidation genes (*atoB* and *fadI*) decrease FA degradation, lowering M-CoA demand. Consistently, CRISPRi knockdowns of *atoB* and other β-oxidation genes (*fadE, fadB* and *yqeF*) have been shown to increase FA levels in *E. coli*^54^.

To assess whether *fadI* similarly prevents FA degradation and increases M-CoA, the BAP1–ΔbioH–MatBC strain was engineered with a *fadI* knockout, creating BAP1–ΔbioH–MatBC–ΔfadI. M-CoA and Ac-CoA levels were compared among this strain, the unevolved BAP1–MatBC strain and the evolved E1_S2 strain carrying a *fadI* mutation. E1_S2 exhibited a substantial increase in M-CoA compared to BAP1–MatBC, whereas BAP1–ΔbioH–MatBC–ΔfadI displayed intermediate M-CoA levels, not significantly different from either strain (Supplementary Fig. 10).

### Characterization of evolved strains

To assess phenotypic differences between clades, one strain from each identified clade (E1_S2, E2_S3, E2_S7 and E2_S17), representing distinct ALE culturing conditions, was cultivated in M9 minimal media with 2% glucose and 10 mM malonate for growth recovery tests (Fig. 5a). In total, 10 mM malonate was chosen as an intermediate concentration between the two cultivation conditions of 1 and 20 mM. Strains from clades 1, 2 and 3 showed substantially improved growth (OD_600_) compared to the unevolved strain, whereas the clade 4 strain exhibited marginally better growth only at early time points. Under its ALE condition (1 mM malonate), clade 4 outperformed the unevolved strain (Supplementary Fig. 8).

To evaluate if evolved strains enhanced M-CoA availability for PKSs and increased polyketide production, the flaviolin production plasmid pBADT–RppA-NT was introduced into one representative strain from each clade (E1_S2, E2_S3, E2_S7 and E2_S17). These strains, along with controls BAP1–ΔbioH–RFP and unevolved BAP1–ΔbioH–MatBC, were cultured under the following three conditions: M9 media + 10 mM malonate, LB and LB + 10 mM malonate. Cultures were grown in quadruplicate at 37 °C for 4 days, and flaviolin production was measured at 520 nm to reduce media background interference between LB and M9 media.

In LB, E2_S7 and E2_S17 produced substantially more flaviolin than other strains, even without malonate. All strains outperformed the RFP control when malonate was added. E1_S2 (clade 4) produced less flaviolin than the unevolved strain, whereas E2_S3, E2_S7 and E2_S17 showed substantially higher production (Fig. 5b). In M9 media, flaviolin production after 24 h was notable only for E1_S2 and E2_S7. However, after 96 h, flaviolin production was observed in all strains, with E2_S3 and E2_S7 outperforming the unevolved strain (Fig. 5c). Owing to its superior performance across conditions, E2_S3 (clade 2) was selected for further study.

To investigate key adaptive mutations in strain E2_S3, its proteome was compared to the unevolved MatBC strain and E1_S2 from clade 4, which had the fewest mutations. All strains were cultivated in M9 minimal medium with 2% glucose and 10 mM malonate for 48 h at 37 °C, starting at an OD_600_ of 0.001. Both evolved strains shared a mutation in the *lac* operator regulating *matB* and *matC* expression and mutations to β-oxidation genes (E2_S3, ∆*atoB;* E1_S2, ∆*fadI*). Proteomic differences were identified using Welch’s *t*-test (cutoff of > ±1-fold, *P* < 0.05), and differentially expressed proteins were annotated by their Clusters of Orthologous Groups (COG) name with the eggNOG-Mapper V2 tool (http://eggnog-mapper.embl.de/)^66^.

Compared to the unevolved strain, E1_S2 showed increased expression of genes associated with amino acid transport and metabolism (E) and nucleotide transport and metabolism (F), with decreased expression of inorganic ion transport genes (P). Notably upregulated proteins included MatB (2.43-fold) and MatC (5.1-fold), along with proteins involved in aromatic amino acid biosynthesis (AroI, 5.7-fold; EntB, 5.4-fold; AroF, 4.8-fold; TyrA, 3.2-fold) and the methylerythritol phosphate pathway (Dxr, 4.0-fold; IspH, 3.4-fold; IspU, 3.4-fold).

In the highly mutated strain E2_S3, compared to the unevolved strain, there was increased expression of genes related to lipid transport and metabolism (I) and decreased expression of genes for signal transduction and nucleotide transport and metabolism (F). Significantly upregulated proteins included MatB (3.9-fold) and MatC (3.6-fold), FA β-oxidation proteins (FadA, FadB, FadD, FadE, FadH, FadI, FadJ, FadM and TesB; 3.9–7.07-fold), glyoxylate shunt proteins (AceA, 2.6-fold; AceB, 5.2-fold) and iron transport proteins (EntC, 5.2-fold; FhuE, 4.4-fold). Decreased expression of TktB, PoxB, PfkB and PurC (−2.82-fold to −1.09-fold) suggests a shift toward the Entner–Doudoroff pathway, which generates NADPH and ATP with fewer enzymatic steps compared to glycolysis and the pentose phosphate pathway. When comparing E2_S3 to E1_S2, the highly mutated E2_S3 showed reduced expression of amino acid (E) and nucleotide transport genes (F) but higher expression of genes related to lipid metabolism (I), inorganic ion transport and secondary metabolite biosynthesis (Q). Additionally, AceA, AceB and MatB were also increased (Fig. 5d).

Enhanced glyoxylate shunt activity enables Ac-CoA to enter the TCA cycle while avoiding carbon loss as CO_2_ and bypassing iron-dependent enzymes. This pathway’s activation is further linked to FA presence, regulated by IclR and indirectly by FadR67. Detailed proteomic and COG data are provided in Supplementary Data 1.

Overall, the proteomic data support the proposed pathways most affected by ALE (Fig. 4c). In E2_S3, mutations in β-oxidation and iron transport likely drive increased expression of related proteins in response to FA accumulation and iron starvation. Increased levels of β-oxidation protein in E2_S3 compared to E1_S2 suggest that *atoB* knockouts more effectively block FA degradation than *fadI* mutations, consistent with intermediate M-CoA levels observed in the reengineered strain. Enhanced glyoxylate shunt activity bypasses carbon loss as CO_2_ and iron-dependent enzymes in the TCA cycle. Additionally, increased expression of the glyoxylate shunt pathway is known to increase in the presence of FAs via negative regulation by IclR and indirectly by FadR^67^. Detailed proteomic and COG data are available in Supplementary Data 1. Additionally, all plasmids and strains created or used in this study are listed in Supplementary Table 1.

## Discussion

Expressing engineered PKSs in *E. coli* is a sustainable approach for producing valuable small molecules. However, unlike native hosts, *E. coli* often lacks sufficient acyl-CoA precursors, including M-CoA, which is tightly regulated and depleted during the stationary phase^18,19^.

This study introduced *matB* and *matC* into PKS-compatible *E. coli* strains (BAP1 and K207-3), enabling precise control over M-CoA levels. This strategy increased titers of M-CoA-derived compounds, including type III PKS naphthoquinones, FAs and type I PKS products. Hybrid type I PKS constructs (pik127-2nd and pik127-3rd acyltransferases) demonstrated how substrate pool variations substantially affect product composition, emphasizing the importance of optimizing acyl-CoA precursor pools.

To further regulate M-CoA, the endogenous M-CoA pathway in BAP1–ΔbioH–MatBC was disrupted by deleting biotin synthesis genes, allowing malonate supplementation to control growth, M-CoA and polyketide titers. This approach achieved up to an 18.1-fold M-CoA improvement over wild-type strains, comparable to or exceeding prior efforts^20^. Leveraging this control over M-CoA facilitated the production of uniformly labeled ^13^C-polyketides without requiring fully isotopically labeled carbon sources. Notably, as most polyketides are derived from a mixture of acyl-CoA starter units, if the PKS encodes for extension by at least one M-CoA, uniform production of the labeled polyketide can be achieved. Compounds enriched with stable isotopes such as ^13^C are invaluable as metabolic tracers in medicine^68,69^ and research, where uniform labeling ensures accurate results^70,71^. Highly ^13^C-enriched compounds are particularly beneficial for in vivo ^13^C MRI trials, where the enhanced signal improves imaging^72–74^. This method offers a cost-effective, efficient alternative for producing >99% uniformly labeled M-CoA-derived products and FAs, outperforming traditional approaches that rely on fully labeled carbon sources like [^13^C]glucose or [^13^C]acetate^75^.

Using the engineered malonate growth dependency, ALE was performed to identify mutations enhancing M-CoA production while avoiding or compensating for mutations that often come at a cost in cellular growth^23,24,76^. The evolved strains acquired many mutations and clustered into four distinct clades. Loss-of-function mutations in genes like *atoB*, *creC* and *glpG* aligned with prior research identifying their roles in enhancing FA or M-CoA titers. More interesting was the presence of loss-of-function mutations to genes that have yet to be targeted through any direct approaches but are in relevant pathways such as β-oxidation, colanic acid biosynthesis, iron transport and amino acid/protein biosynthesis, where knockouts or knockdowns of genes have previously been shown to improve FA, M-CoA or polyketide titers. Although some of our observed mutations could be rationalized, many remain unclear. Re-engineering these mutations, particularly to transcription factors (*abgR*, *dmlR*, *ttdR*, *treR* and *rhaR*), could further elucidate their effects.

Finally, the evolved strains were assessed for M-CoA-derived polyketide production. Evolved strains were identified that outperformed the original strains in minimal medium with malonate and in biotin-rich conditions (for example, LB), indicating mutations that enhance M-CoA availability even outside selective conditions. This highlights ALE as a powerful tool for improving M-CoA flux and polyketide biosynthesis while uncovering pathways involved in FA and CoA metabolism.

Regulating M-CoA pools is essential for the biosynthesis of polyketides derived from this precursor. Orthogonal pathways that boost M-CoA levels provide opportunities to investigate, engineer and evolve M-CoA metabolism for improved heterologous PKS expression in *E. coli*. Using ALE to identify mutations that increase flux toward M-CoA and downstream PKS products highlights the utility of biotin/malonate auxotrophic strains. Moreover, ALE facilitates the selection of compensatory mutations that can alleviate growth defects while also enhancing M-CoA production, further supporting the evolution of robust strains for polyketide biosynthesis.

## Methods

### Chemical compounds and standards

All chemicals, reagents and solvents were purchased from commercial suppliers and used without further purification unless otherwise stated. Standard laboratory chemical reagents were obtained from Sigma-Aldrich or Thermo Fisher Scientific. Water used in all experiments was purified using a Milli-Q system. Specific sources for specialized reagents or specific solutions are listed below:

1. The 2.5 M stock solution of [^13^C]malonic acid (Santa Cruz Biotechnology) was prepared by the addition of NaOH to neutral pH.
2. The 2.5 M stock solution of disodium malonate (Spectrum Chemicals) was prepared in Milli-Q H_2_O and adjusted to a neutral pH.
3. M9 minimal medium—1× M9 salts, 2% glucose, 2 mM MgSO_4_, thiamine (1 µg ml^−1^), 0.1 mM CaCl_2_ and filter sterilized.
4. 6-Ethyl-3,5-dimethyldihydro-2H-pyran-2,4(3H)-dione was prepared as previously reported^77^.
5. 6-Ethyl-5-methyldihydro-2H-pyran-2,4(3H)-dione was prepared as previously reported^78^.

### Cloning of expression plasmids

*matB* and *matC* were PCR-amplified from the plasmid pCKmatBC purchased from Addgene (138587)^39^. PCR reactions were performed using Phusion High-Fidelity DNA Polymerase 2X Master Mix (New England Biolabs (NEB)) or PrimeSTAR Max DNA Polymerase 2× Master Mix (Takara). Gibson Assembly was performed using NEBuilder HiFi DNA Assembly to assemble the plasmids—pBbS5k_pik12(pik_AT3), pBbA5a_pik27, pR6K–ompW–MatBC, pR6K–bioH–RFP and pR6K–bioH–matBC. QuikChange Site-Directed Mutagenesis Kit was used to create the pR6K–ompW–RFP* vector without an NdeI cut site in the backbone, allowing for NdeI/XhoI insertion of genes of interest by restriction digest and T4 ligation. Plasmids pBbA2c–MatB–MadLM and pBbA2c–MatB–MdcF were constructed by digesting the BglBrick vector pBbA2c_RFP^79^ with EcoRI and BamHI. *E. coli*-optimized sequences for the *mdcF* and *madL* and *madM* genes were ordered as gene fragments from Integrated DNA Technologies (IDT) with overhang regions encoding ribosomal binding site (RBS) sequences and EcoRI and BamHI restriction sites. Gene inserts were also restriction-digested and assembled using standard T4 ligation protocols. Subsequently, these intermediate plasmids were sequence-verified and then digested with EcoRI and BglII. The *E. coli* codon-optimized *matB* was also ordered, as well as a gene fragment from IDT with an RBS sequence and corresponding EcoRI and BglII restriction digest sites. The *matB* fragment was also restriction-digested and assembled with the *madLM* and *mdcF* backbones using standard T4 ligation protocols. All plasmids and strains used in this study are listed in Supplementary Table 1. Gene-specific primers are listed in Supplementary Table 2. Specific gene sequences are listed in Supplementary Table 3. Restriction enzymes were used as recommended by the manufacturer (NEB). Assembled plasmids containing the R6K backbone were transformed into One Shot Pir2 chemically competent cells (Thermo Fisher Scientific). All other plasmids were transformed into XL1-Blue or NEB 5-α F′*Iq* chemically competent cells. Single colonies of the transformants were cultured and miniprepped using the QIAprep Spin Miniprep Kit. All the plasmid sequences were validated by whole-plasmid sequencing from Primordium Labs.

### Integration of DNA into *E. coli*

Strains BAP1 and K207-3 were transformed with the temperature-sensitive red recombinase plasmid pKD46 (ref. ^80^) and cultured in LB medium with carbenicillin (100 µg ml^−1^) at 30 °C. Fresh pKD46 transformants were grown to OD_600_ ~0.5, induced with 0.2% arabinose for 1 h and made electrocompetent by 100-fold concentration and three washes with ice-cold 10% glycerol.

pR6K plasmids containing homology arms and the gene of interest were PCR-amplified using integration primers (Supplementary Table 2), gel-purified (Zymo Research) and eluted in 10 µl nuclease-free water. Approximately 500 ng of PCR product was electroporated into cold electrocompetent cells in a 0.1 cm gap disposable cuvette using a Bio-Rad Micropulser (settings—EC1, 1.8 kV) and rescued with 1 ml SOC (Super Optimal broth with catabolite repression) medium at 30 °C for 2 h. The culture was concentrated to 100 µl, plated on LB + chloramphenicol (30 µg ml^−1^) and then incubated overnight at 30 °C. Transformants were screened by colony PCR with screen primers (Supplementary Table 2), and positive clones were cultured at 37 °C to remove pKD46.

Antibiotic resistance genes flanked by flippase recognition target sites were excised using the temperature-sensitive flip recombinase plasmid PCP20 (ref. ^80^). Transformants were plated on LB + carbenicillin (100 µg ml^−1^), grown overnight at 30 °C, and screened via colony PCR. Successful excision was confirmed by sequencing and restored sensitivity to chloramphenicol (30 µg ml^−1^). PCP20 was removed by restreaking on LB and incubating at 42 °C overnight.

For deletion of *fadI* and biotin operon genes (*bioA*, *bioB*, *bioC*, *bioD* and *bioF*), pKD13 was amplified with FadI-K/O or Bio-K/O primers to generate dsDNA encoding kanamycin resistance flanked by the corresponding homology arms. PCR products were gel-purified and eluted in 10 µl nuclease-free water. pKD46 transformants of BAP1–∆bioH–MatBC were grown to OD_600_ ~0.5, induced with 0.2% arabinose for 1 h and made electrocompetent by 100-fold concentration and three washes with ice-cold 10% glycerol. Approximately 500 ng of PCR product was electroporated, and cells were rescued in SOC at 30 °C for 2 h. Transformants were screened via colony PCR, and positive clones were cultured at 37 °C to remove pKD46. Antibiotic resistance cassettes were excised using PCP20 as detailed above.

### Screening of malonate importers using plasmid-based systems

Electrocompetent BAP1 cells were transformed with plasmids pCKmatBC, pBbA2c–MatB–MadLM and pBbA2c–MatB–MdcF via electroporation (Micropulser; EC1, 1.8 kV). Cells were rescued in SOC (1 ml, 1 h, 37 °C), plated on LB + chloramphenicol (30 µg ml^−1^) and then incubated overnight at 37 °C. The pBADT–RppA-NT plasmid carrying a truncated *rppA* sequence from *S. coelicolor* for increased enzymatic activity was transformed into BAP1 strains with malonate importer plasmids using the same electroporation protocol^44,81^. Transformants were plated on LB + chloramphenicol (30 µg ml^−1^) + kanamycin (50 µg ml^−1^) and incubated overnight at 37 °C

Initial attempts to induce the MadLM- and MdcF-containing strains with 10 nM aTc resulted in severe growth defects. As such, only the MatBC plasmid was induced with the addition of IPTG (10 µM) to the media. Cultures were grown in LB + 0.2% arabinose + 10 µM IPTG with 0, 1, 5 or 10 mM malonate. Absorbance at 340 nm was monitored over 3 days. Triplicate cultures were grown in 24-well deep-well plates (30 °C, 200 rpm). Aliquots (200 µl) were collected at 24, 48 and 72 h and centrifuged (5,000*g* for 10 min), and 100 µl supernatant was transferred to a 96-well black clear-bottom plate for absorbance measurement (340 nm and 520 nm; Biotek Synergy 4 Plate Reader). Supernatants were diluted as needed to remain within the linear detection range (Supplementary Fig. 1).

### Screening of integrated MatBC strains in LB and M9 media

The pBADT–RppA-NT plasmid was transformed into BAP1–∆bioH–RFP, K207-3, BAP1–ΔbioH–MatBC and K207-3–MatBC strains, with selection on kanamycin (50 µg ml^−1^). Colonies were inoculated into LB + kanamycin (50 µg ml^−1^) and grown overnight at 37 °C. Cultures were diluted 1:100 into fresh LB or M9 minimal media with kanamycin (50 µg ml^−1^), 0.2% l-arabinose, 10 µM IPTG and increasing malonate concentrations (0–20 mM). Triplicate cultures were grown in 24-well deep-well plates (30 °C at 200 rpm). Aliquots (200 µl) were collected at 24, 48 and 72 h and centrifuged (5,000*g* for 10 min), and 100 µl of supernatant was transferred to a 96-well black clear-bottom plate for absorbance measurement (340 nm and 520 nm; Biotek Synergy 4). Samples were diluted as needed to remain within the linear detection range.

To quantify flaviolin concentration, LB blank absorbance was subtracted from samples, and extinction coefficients (*ε*_340_ = 3,068 M^−1^ cm^−1^ and *ε*_520_ = 1,305 M^−1^ cm^−1^) were applied for approximation^82^.

### Flaviolin production in M9 minimal medium with malonate or [^13^C]malonate

A single BAP1–ΔbioH–MatBC colony carrying pBADT–RppA-NT was inoculated into LB + kanamycin (50 µg ml^−1^) and grown overnight at 37 °C. The culture (1 ml) was centrifuged (13,000*g*), washed three times with M9 buffer, resuspended in 1 ml of M9 buffer and diluted 1:1,000 into 10 ml of fresh M9 medium containing 2% glucose, 0.2% l-arabinose, 10 µM IPTG and kanamycin (50 µg ml^−1^).

Aliquots (2 ml) were distributed into glass tubes and supplemented with the following five conditions: (1) control (no malonate/biotin), (2) 20 mM malonate, (3) 20 mM [^13^C]malonate, (4) 25 mg l^−1^ biotin and (5) 20 mM [^13^C]malonate + 25 mg l^−1^ biotin. Cultures were incubated at 37 °C for 3 days, with 50 µl samples taken at 24, 48 and 72 h for LC–MS analysis.

Notably, for condition 1 (no malonate/biotin), the strain does not grow, whereas the strains supplemented with biotin or malonate turn from light pink on day 1 to visibly turbid and dark red by day 3 (Supplementary Fig. 5). OD_600_ and flaviolin production were determined for the final 72 h time point by measuring the absorbance at 600 nm and 340 nm using a path length of 0.1 cm^−1^.

For LC–MS analysis, samples were mixed with 50 µl LC–MS grade methanol + 0.1% formic acid and centrifuged (13,000*g*), and the supernatant was further purified using Amicon Ultra-0.5 centrifugal filters (3 kDa, 14,000*g* for 15 min), and the flow through was then transferred to LC–MS vials.

Samples were analyzed by a 1260 Infinity High Performance Liquid Chromatography (HPLC) system (Agilent Technologies) coupled to an Agilent LC–MSD and a 6520 Quadrupole Time-of-Flight Mass Spectrometer system (Agilent Technologies). In total, 10 µl of the sample was injected onto a Phenomenex Kinetex XB-C18 (2.6 µm, 100 × 3 mm, 100 Å) LC column and separated with the following HPLC protocol: mobile phase A was composed of 0.1% formic acid in water, and mobile phase B was composed of 0.1 % formic acid in methanol. The QTOF–MS acquisition parameters were as follows: drying and nebulizing gases were set to 11 l min^−1^ and 30 lb in^−2^, respectively, and a drying-gas temperature of 330 °C. ESI was conducted in the negative ion mode with a capillary voltage of 3,500 V, scanning from 100 to 300 *m*/*z*. Data acquisition (Workstation B.08.00) and processing were conducted using the Agilent MassHunter software package.

### Flaviolin production in evolved strains (M9 minimal medium and LB media)

Single colonies of BAP1–ΔbioH–RFP and BAP1–ΔbioH–MatBC and the evolved strains E1_S2, E2_S3, E2_S7 and E2_S17 (kanamycin resistance removed via pcp20-mediated FLP recombination) were transformed with pBADT–RppA-NT and selected on kanamycin. Colonies were inoculated into LB + kanamycin (50 µg ml^−1^) and grown overnight at 37 °C.

Cultures (1 ml) were centrifuged (13,000*g*), washed three times, resuspended in 1 ml M9 minimal medium and then diluted 1:1,000 into four replicates in 24-well deep-well plates. Cultures were incubated at 37 °C, 200 rpm in the following:

1. M9 minimal medium + 10 mM malonate, 2% glucose, 0.2% l-arabinose, 1× trace elements and 50 µg ml^−1^ kanamycin.
2. LB + 0.2% l-arabinose and 50 µg ml^−1^ kanamycin.
3. LB + 10 mM malonate, 0.2% l-arabinose and 50 µg ml^−1^ kanamycin.

Cultures were shaken at 37 °C, 200 rpm for 4 days, with 50 µl supernatant samples taken every 24 h, diluted 1:1 with Milli-Q H_2_O and transferred to a 96-well black clear-bottom plate for absorbance measurement (340 nm and 520 nm; Biotek Synergy Plate Reader). Samples exceeding the linear detection range were further diluted. To quantify flaviolin concentration, the media blank absorbance was subtracted. Absorbance at 520 nm was used instead of 340 nm to correct for background differences between LB and M9 minimal media.

### Pikromycin hybrid PKS fermentation

For the pik127 PKSs, the three-module PKS is split across two plasmids. For the hybrid PKS construct that has the M-CoA loading at the first extension module, the combination of the plasmids pBbS5k_pik12(pik_AT3) and pBbA5a_pik27 in the K207-3 and K207-3–MatBC results in the strains KCP9 and KCP9–MatBC. For the hybrid PKS construct that has the M-CoA loading at the second extension module, the combination of the plasmids pBbS5k_pik12 and pBbA5a_pik27(pik_AT3) in the K207-3 and K207-3–MatBC strains is also reported in ref. ^78^ to create KCP3 and KCP3–MatBC.

Dual-plasmid strains (KCP3, KCP3–MatBC, KCP9 and KCP9–MatBC) were selected on LB + spectinomycin and kanamycin (50 µg ml^−1^). Colonies were grown overnight in LB + carbenicillin (100 µg ml^−1^) and kanamycin (50 µg ml^−1^) at 30 °C and then diluted 1:100 into 2 ml fresh EZ-rich medium with the same antibiotics. Triplicate cultures for each condition were incubated in 24-well deep-well plates at 37 °C, 200 rpm.

At OD_600_ ~0.6, cultures were cooled to 18 °C and induced with 0.1 mM IPTG. Upon induction, 20 mM propionate was added to all wells, and 20 mM malonate was added in experimental conditions with corresponding no-malonate controls. Fermentations were run in triplicate. After 6 days (18 °C, 200 rpm), plates were frozen at −80 °C for further analysis.

Frozen cultures were thawed, and 200 µl was transferred to a 96-well plate containing 200 µl of 10 µM internal standard TAL in acetonitrile (ACN) and 2 µl concentrated HCl. Compounds were extracted by incubating at 37 °C, 200 rpm for 30 min. Cell debris was removed by centrifugation (5,000*g* for 20 min), and supernatants were filtered using Amicon Ultra-0.5 centrifugal filters (3 kDa, 14,000*g* for 15 min). The flow through was then transferred to LC–MS vials and analyzed using a 1260 Infinity HPLC system (Agilent Technologies) coupled to an Agilent Liquid Chromatography Mass Spectrometry Detector (LC–MSD) and a 6520 Quadrupole Time-of-Flight Mass Spectrometer (QTOF-MS) system (Agilent Technologies). In total, 10 µl of the sample was injected onto a Phenomenex Kinetex XB-C18 (2.6 µm, 100 × 3 mm, 100 Å) LC column and separated with the following HPLC protocol: mobile phase A was composed of 0.1% formic acid in water, and mobile phase B was composed of 0.1% formic acid in methanol. The QTOF–MS acquisition parameters were as follows: drying and nebulizing gases were set to 11 l min^−1^ and 30 lb in^−2^, respectively, and a drying-gas temperature of 330 °C. ESI was conducted in the negative ion mode with a capillary voltage of 3,500 V, scanning from 100 to 300 *m*/*z*. Data acquisition (Workstation B.08.00) and processing were conducted using Agilent MassHunter software package.

### Free FA quantification

BAP1–∆bioH–RFP and BAP1–ΔbioH–MatBC cultures were grown in M9 minimal medium with 2% glucose, 10 µM IPTG and 20 mM malonate in 24-well plates (2.5 ml, 37 °C, 200 rpm, 20 h). For BAP1–∆bioH–RFP, 25 mg l^−1^ biotin was added. All strains and conditions were tested in triplicate, with the experiment performed twice (*n* = 6).

For sample preparation, 100 µl of culture was first removed to measure OD_600_ for cell density determination. Then, 500 µl of culture was spiked with 5 µl of 25 mM nonanoic acid as an internal standard (final concentration—250 µM), followed by acidification with 100 µl glacial acetic acid. After vortexing, 2 ml of ethyl acetate was added for FA extraction, and samples were incubated for 30 min at 200 rpm. Following centrifugation at 4,000*g* for 10 min, the organic layer was transferred to a screw-top tube and left to evaporate overnight in a fume hood.

To derivatize the extracted FAs, 200 µl of 1.25 M anhydrous HCl in methanol was added to each dried sample. Tubes were tightly capped and incubated at 80 °C for 1 h. After cooling, 500 µl of hexane was added, and samples were vortexed before centrifugation at 15,000*g* for 1 min. The hexane layer was collected for GC–MS analysis.

For detection, 2 µl of the sample was injected using a CTC Analytics PAL System automated sampler onto an Agilent 5973 Network Mass Selective Detector GC–MS. The GC temperature program was as follows: initial *T* = 50 °C, ramp 10 °C min^−1^ to 160 °C, ramp 30 °C min^−1^ to 230 °C hold 1 min, ramp to 300 °C hold 3 min, return to 50 °C. MS acquisition parameters were as follows: mass detection range, *m*/*z* = 50–350 Da.

EICs of *m/z* 74 Da, corresponding to the McLafferty rearrangement ion of FA methyl esters, were used to identify peaks. FA peaks were integrated and quantified using calibration curves for C12, C14, C16 and C18 FAs, with nonanoic acid as the internal standard.

### Proteomics analysis

Four biological replicate cultures of BAP1–ΔbioH–MatBC, BAP1–ΔbioH–MatBC–ΔbioH–kan, E1_S2 and E2_S3 were grown in 2 ml M9 minimal medium with 2% glucose and 10 mM malonate. All strains were cultivated in M9 minimal medium with 2% glucose and 10 mM malonate for 48 h at 37 °C after inoculation at an OD_600_ of 0.001. In total, 1 ml of cell culture was collected by centrifugation at 5,000*g* for 15 min, and the resulting cell pellets were stored at −80 °C until further processing.

Protein was extracted from cell pellets, and tryptic peptides were prepared by following the established proteomic sample preparation protocol^83^. Briefly, cell pellets were resuspended in Qiagen P2 Lysis Buffer (Qiagen) to promote cell lysis. Proteins were precipitated with the addition of 1 mM NaCl and 4× vol acetone, followed by two additional washes with 80% acetone in water. The recovered protein pellet was homogenized by pipetting and mixing with 100 mM ammonium bicarbonate in 20% methanol. Protein concentration was determined by the detergent compatible (DC) protein assay (Bio-Rad). Protein reduction was accomplished using 5 mM tris 2-(carboxyethyl)phosphine for 30 min at room temperature, and alkylation was performed with 10 mM iodoacetamide (final concentration) for 30 min at room temperature in the dark. Overnight digestion with trypsin was performed at a 1:50 trypsin-to-total protein ratio. The resulting peptide samples were analyzed on an Agilent 1290 Ultra High Performance Liquid Chromatography (UHPLC) system coupled to a Thermo Scientific Orbitrap Exploris 480 mass spectrometer for discovery proteomics^84^. Briefly, peptide samples were loaded onto an Ascentis ES-C18 Column (Sigma-Aldrich) and were eluted from the column by using a 10-min gradient from 98% solvent A (0.1 % FA in H_2_O) and 2% solvent B (0.1 % FA in ACN) to 65% solvent A and 35% solvent B. Eluting peptides were introduced to the mass spectrometer operating in positive-ion mode and were measured in data-independent acquisition (DIA) mode with a duty cycle of three survey scans from *m*/*z* 380 to *m*/*z* 985 and 45 MS2 scans with precursor isolation width of 13.5 *m*/*z* to cover the mass range. DIA raw data files were analyzed by an integrated software suite, DIA-NN^85^. The database used in the DIA-NN search (library-free mode) is *E. coli*’s latest UniProt proteome FASTA sequences plus the protein sequences of the heterologous proteins and common proteomic contaminants. DIA-NN determines mass tolerances automatically based on first-pass analysis of the samples with automated determination of optimal mass accuracies. The retention time extraction window was determined individually for all MS runs analyzed via the automated optimization procedure implemented in DIA-NN. Protein inference was enabled, and the quantification strategy was set to robust LC = high accuracy. The output main DIA-NN reports were filtered with a global false discovery rate (FDR) = 0.01 on both the precursor level and protein group level. The Top3 method, which is the average MS signal response of the three most intense tryptic peptides of each identified protein, was used to plot the quantity of the targeted proteins in the samples^86,87^.

The mass spectrometry proteomics data have been deposited in the ProteomeXchange Consortium via the PRIDE^88^ partner repository with the dataset identifier PXD060741. DIA-NN is freely available for download from https://github.com/vdemichev/DiaNN.

Significant differences between the proteomic profiles of the strains were determined by using a Welch’s *t*-test and a cutoff of > ±1-fold and a *P* value of <0.05. Significantly up and down proteins were then annotated when function could be identified using their COG name with the eggNOG-Mapper V2 tool (http://eggnog-mapper.embl.de/)^66^. Excel files containing all the significantly up and down proteins for each comparison are provided in Supplementary Data 1.

### Quantification of M-CoA and Ac-CoA

Cultures of BAP1 and BAP1–ΔbioH–MatBC were grown in 3.5 ml of M9 minimal medium supplemented with 2% glucose and 10 µM IPTG, with increasing concentrations of malonate (0–100 mM) prepared from a 2.5 M sodium malonate dibasic stock solution. Each condition was tested in triplicate using 24-well deep-well plates covered with breathable film. Cultures were incubated at 37 °C with shaking at 200 rpm for 36 h. After incubation, 25 µl of culture was removed, diluted fourfold and OD_600_ was measured and converted to DCW using the factor 1 OD_600_ = 0.396 g DCW^89^. The remaining culture was centrifuged at 4 °C for 10 min to pellet cells. The supernatant was collected for HPLC analysis of malonate, glucose and acetate concentrations, while the cell pellet was frozen at −80 °C for later analysis. Before LC–MS analysis, frozen cell pellets were thawed on ice. To each pellet, 350 µl of a 45:45:10 ACN:MeOH:H_2_O solution containing 0.1% glacial acetic acid was added. Cells were resuspended by repetitive pipetting, followed by centrifugation at 13,000*g* at 4 °C for 15 min. A 100 µl aliquot of the supernatant was transferred to LC–MS vials for subsequent analysis, where 10 µl of sample was injected onto a C18 (2.6 µm, 100 × 3 mm, 100 Å) LC column, and analyzed with the following HPLC protocol: buffer A—water with 20 mM ammonium acetate; buffer B—MeOH with 20 mM ammonium acetate; flow rate—0.60 ml min^−1^; 2% buffer B for 1 min, 2–85% buffer B gradient for 3.0 min, 85–90% buffer B gradient for 2.0 min, 90–95% buffer B gradient for 2.0 min, 95–2% buffer B for 0.2 min, re-equilibration at 2% buffer B for 4.8 min. MS acquisition parameters were as follows: mass-to-charge ratios corresponding to 854 Da (M-CoA) and 810 Da (Ac-CoA) were monitored in SIM positive mode.

### ALE of BAP1–ΔbioH–MatBC

#### One-week mutagenesis turbidostat

The BAP1–ΔbioH–MatBC strain was transformed with the mutagenesis-enhancing plasmid *MP6*, and transformants were selected on LB agar containing chloramphenicol (30 µg ml^−1^). A single colony was grown overnight in M9 minimal medium with 2% glucose and 10 mM malonate. The culture was then diluted 1:100 into fresh M9 minimal medium supplemented with 2% glucose, 30 µg ml^−1^ chloramphenicol, 1% arabinose and 1 mM malonate. The culture was maintained in a Chi.Bio turbidostat at an OD_600_ of 0.75 through continuous dilution over 7 days^52^.

On day 7, an aliquot of the evolved strain was streaked onto M9 minimal agar plates containing 3 mM malonate and incubated at 37 °C. Individual colonies were subsequently inoculated into LB, M9 minimal medium and M9 minimal medium with 1 mM malonate and grown at 37 °C for 24 h. Mutants were identified by their inability to grow in M9 minimal medium alone but increased growth in malonate-supplemented conditions. The genomes of six mutants with the highest OD_600_ values (E1_S1–S6) were selected for sequencing.

#### Four-week mutagenesis serial dilution

The BAP1–ΔbioH–MatBC–Δbio-kan strain was transformed with MP6, and transformants were isolated on LB agar with chloramphenicol (30 µg ml^−1^). A single colony was grown overnight in M9 minimal medium supplemented with 2% glucose, 50 µg ml^−1^ kanamycin and 20 mM malonate. The culture was then diluted 1:100 into 5 ml of fresh M9 medium containing 2% glucose, 30 µg ml^−1^ chloramphenicol, 50 µg ml^−1^ kanamycin, 1% arabinose and 20 mM malonate in a 30 ml glass culture tube. The culture was maintained through serial dilution over 14 days.

On day 14, an aliquot was saved as a glycerol stock, streaked onto kanamycin (50 µg ml^−1^) agar plates and incubated at 37 °C. The remaining culture was split into two new tubes containing fresh M9 minimal medium with 2% glucose, 30 µg ml^−1^ chloramphenicol, 50 µg ml^−1^ kanamycin and 1% arabinose, with either 1 mM or 20 mM malonate added. These cultures were maintained through serial dilution for an additional 14 days.

At the end of the experiment (day 28), aliquots of evolved strains were saved as glycerol stocks and streaked onto kanamycin agar plates (50 µg ml^−1^). Individual colonies from the three plates were inoculated into M9 minimal medium supplemented with 10 mM malonate and grown at 37 °C for 24 h. A total of 17 colonies (E2_S1–S17) were selected for whole-genome sequencing.

### Whole-genome sequencing

#### Samples from 1-week mutagenesis turbidostat

Using the method adapted from ref. ^90^, genomic DNA was prepared from cultured strains using a Wizard Genomic DNA Purification Kit. In total, 100 ng of DNA was sheared to 600 bp using the fragmentation enzyme in the xGen DNA Lib Prep EZ Kit (Integrated DNA Technologies), and size was selected using SPRI beads (Beckman Coulter). The fragments were treated with end-repair, A-tailing and ligation of Illumina-compatible adapters (Integrated DNA Technologies) using the xGen DNA Lib Prep EZ Kit. Bioanalyzer High Sensitivity DNA Kit (Agilent) and Qubit Fluorometers (Thermo Fisher Scientific) were used to determine the concentration of the libraries. Libraries were sequenced on the Illumina MiSeq.

#### Samples from 2- to 4-week mutagenesis serial dilution

Overnight cultures were diluted to 4–6 × 10^9^ cells per ml and then centrifuged at 13,000*g* to pellet the cells. The cell pellet was washed once in 1 ml of 1× PBS and resuspended in 500 μl of Zymo 1× DNA/RNA Shield preservative and submitted for bacterial whole-genome sequencing performed by Plasmidsaurus using Oxford Nanopore Technology with custom analysis and annotation.

### Hierarchical clustering and dendrogram construction

To analyze the genetic relationships between the 23 *E. coli* strains based on their mutation profiles, a hierarchical clustering analysis was performed using a presence–absence mutation matrix. In this binary matrix, rows represented specific mutations and columns corresponded to individual strains, with a value of 1 indicating the presence of a mutation and 0 indicating its absence.

For data processing, the mutation matrix was transposed so that each strain was represented as a binary vector corresponding to its mutation profile. Hierarchical clustering was conducted on this transposed matrix using the Morpheus online platform (https://software.broadinstitute.org/morpheus)^91^. The dendrogram was calculated using one minus the Pearson correlation and an average linkage distance.

### Structure prediction

The sequence for MatC was trimmed at the N and C termini to remove disordered regions. AlphaFold 2.3 was used to predict the protein structure of MatC monomers using the configuration scheme in Table 1. All models were run with refinement disabled and number of recycles set to 1. All five default models from the 128 random seeds predicted were used in data analysis, totaling 640 structures. A pairwise root mean square deviation (RMSD) matrix was constructed using alignments through the PyMOL Python package. A two-dimensional projection of the matrix was constructed using multidimensional scaling and transformed using principal components analysis (PCA) with the Scikit-learn Python package. Points were clustered using the DBSCAN algorithm, and representative cluster control models were selected via a script identifying structures with the most similarity to all others within their respective clusters (Supplementary Fig. 8).

**Table 1:** AlphaFold 2 configurations for predicting MatC conformations

Group	max_msa	max_extra_msa	Dropout enabled	Random seeds
1	16	32	Yes	16
2	16	32	No	16
3	32	64	Yes	16
4	32	64	No	16
5	64	128	Yes	16
6	64	128	No	16
7	256	512	Yes	16
8	256	512	No	16

All visualization was done with Python scripts through PyMOL (the PyMOL Molecular Graphics System, v.3.0; Schrödinger).

### Reporting summary

Further information on research design is available in the Nature Portfolio Reporting Summary linked to this article.

## Online content

Any methods, additional references, Nature Portfolio reporting summaries, source data, extended data, supplementary information, acknowledgements, peer review information; details of author contributions and competing interests; and statements of data and code availability are available at 10.1038/s41589-025-01911-6.

## Supplementary information

Supplementary Information Supplementary Tables 1–3 and Supplementary Figs. 1–10.

Reporting Summary

Supplementary Data 1 Full proteomic dataset used to create volcano plots in Fig. 5.

Supplementary Data 2 Full ALE amino acid mutations list per strain and absence–presence mutation matrix of frameshift and stop codon mutations.

## Source data

Source Data Fig. 1 Exact value of each data point.

Source Data Fig. 2 Exact value of each data point.

Source Data Fig. 3 Exact value of each data point.

Source Data Fig. 5 Exact value of each data point.

## Data Availability

The mass spectrometry proteomics data have been deposited in the ProteomeXchange Consortium via the PRIDE^88^ partner repository with the dataset identifier PXD060741. DIA-NN is freely available for download from https://github.com/vdemichev/DiaNN. All strains and their corresponding registry IDs used in this study are listed in Supplementary Table 1 and are available through the Joint BioEnergy Institute’s Inventory of Composable Elements, an open-source registry software and platform for managing information about biological parts (https://public-registry.jbei.org/folders/894). Additionally, whole-genome sequencing files of the evolved mutants are linked to the corresponding strains. Data are available from the corresponding authors upon request. Source data are provided with this paper.
