# Supplementary material for: Engineering controllable alteration of malonyl-CoA levels to enhance polyketide production
Source: Nat Chem Biol. 2025 Jun 11;21(8):1214–25. doi: 10.1038/s41589-025-01911-6 (PMC12303837; doi:10.1038/s41589-025-01911-6)
Supplement: Supplementary file 1 — Supplementary Tables 1–3 and Supplementary Figs. 1–10. [file 41589_2025_1911_MOESM1_ESM.pdf]

nature chemical biology

Supplementary information

https://doi.org/10.1038/s41589-025-01911-6

Engineering controllable alteration of malonyl-CoA levels to enhance polyketide production

In the format provided by the authors and unedited

Table of Contents

Supplementary Table 1. Plasmids and strains used in this study……….…………………………………….2
Supplementary Table 2. Primers used in this study ………………………………..….……………..…...6
Supplementary Table 3. Genes used in this study ……………………………………………………..7
Supplementary Figure 1. ………………………………………………………………….....................8
Supplementary Figure 2. ………………………………………………………………….................9
Supplementary Figure 3. ……………………………………………….…………….….…………10
Supplementary Figure 4. ………………………………………………..………………………..11
Supplementary Figure 5 ……………………………………………………..…………………...12
Supplementary Figure 6. …………………………………………………………..………………..12
Supplementary Figure 7..………………………………………………………………..…………..13
Supplementary Figure 8….………………………………………………………………...........13
Supplementary Figure 9…………………………………………………...………………………...14
Supplementary Figure 10…..…...……………………………………………...……………………15
Supplementary References ……….……………………………………………...……………………16

Supplementary Table 1. Plasmids and strains used in this study		
**Strains and plasmids**	**Description**	**Source or reference**
**Plasmids**		
pCKmatBC	Plasmid carrying the genes for *matB* and *matC* from *R. trifolii*	Addgene: #138587 Jeschek et al. 2017[1]
pBbA2c_RFP	Plasmid used for the construction of *madLM* and *mdcF* containing transporters	[2]
pBbA2c-MatB-MadLM	Plasmid carrying the genes for *madLM* malonate importer	This work
pBbA2c-MatB-MdcF	Plasmid carrying the genes for *mdcF* malonate importer	This work
pBADT-rppA-NT	Plasmid carrying the genes for *rppA* type III PKS	Incha et al. 2020[3]
MP6	Mutagenesis plasmid	[4]
pR6Kan-BioH-RFP	R6Kan vector carrying genes for *rfp* with flanking bioH integration sites	This work
pR6Kan-BioH-MatBC	R6Kan vector carrying genes for *matB* and *matC* with flanking bioH integration sites	This work
pR6Kan-ompW-RFP*	R6Kan vector with flanking homology arms for integration at the intergenic region next to *ompW* with single point mutation in backbone to allow for single flanking NdeI XhoI cut sites at *rfp*	This work
pR6Kan-ompW-MatBC	R6kan vector carrying genes for *matB* and *matC* with flanking homology arms for integration at the intergenic region next to *ompW*	This work
pBbS5k_pik12	Plasmid carrying the first half of the *pik127* hybrid PKS	[5]
pBbA5a_pik27(pik_AT3)	Plasmid carrying the second half of the *pik127* hybrid PKS with the Pik module 3 AT in the 3rd module	[5]
pBbS5k_pik12(pik_AT3)	Plasmid carrying the first half of the *pik127* hybrid PKS with the Pik module 3 AT in the second module	This work
pBbA5a_pik27	Plasmid carrying the second half of the *pik127* hybrid PKS	This work
pKD13	Plasmid for creating knockout strains via homologous recombination	[6]
**Strains**		
XL1Blue	Chemically competent cloning strain	QB3 MacroLab (UC Berkeley)
Pir2-R6Kan-BioH-RFP	Pir2 strain harboring the R6Kan-BioH-RFP plasmid	This work JBEI registry: JBEI- 266065
Pir2-R6Kan-BioH-MatBC	Pir2 strain harboring the R6Kan-BioH-MatBC plasmid	This work JBEI registry: JBEI- 266064
Pir2-R6Kan-ompW-MatBC	Pir2 strain harboring the R6Kan-ompW-MatBC plasmid	This work JBEI registry:

		JBEI-266066
KCP3	K207-3 strain harboring the dual plasmid system to produce pik127-3rdAT: pBbS5k_pik12 + pBbA5a_pik27(pik_AT3)	(Wang et al. 2023)[5]
KCP9	K207-3 strain harboring the dual plasmid system to produce pik127-2ndAT: pBbS5k_pik12(pik_AT3) + pBbA5a_pik27	This work<br>JBEI registry:<br>JBEI-238893
K207-3-MatBC-KCP3	K207-3-MatBC strain harboring the dual plasmid system to produce pik127-3rdAT: pBbS5k_pik12 + pBbA5a_pik27(pik_AT3)	(Wang et al. 2023)[5]
K207-3-MatBC-KCP9	K207-3-MatBC strain harboring the dual plasmid system to produce pik127-2ndAT: pBbS5k_pik12(pik_AT3) + pBbA5a_pik27	This work<br>JBEI registry:<br>JBEI-266038
BAP1	Bl21(DE3) derivative with Bacillus subtilis sfp gene integrated	[7]
K207-3	BAP1 derivative with *S. coelicolor* PCC genes, accA1 and pccB integrated	[8]
BAP1-pMatB-MadLM-RppA	BAP1 strain harboring the pBbA2c-MatB-MadLM plasmid and the pBADT-rppA-NT plasmid	This work<br>JBEI registry:<br>JBEI-265986
BAP1-pMatB-MdcF-RppA	BAP1 strain harboring the pBbA2c-MatB-MdcF plasmid and the pBADT-rppA-NT plasmid	This work<br>JBEI registry:<br>JBEI-265987
BAP1-pMatBC-RppA	BAP1 strain harboring the MatBC plasmid and the pBADT-rppA-NT plasmid	This work<br>JBEI registry:<br>JBEI-265988
BAP1-ΔbioH-RFP	E. coli BAP1 strain with RFP genes integrated at bioH site	This work<br>JBEI registry:<br>JBEI-265989
BAP1-ΔbioH-MatBC	E. coli BAP1 strain with MatBC genes integrated at bioH site	This work<br>JBEI registry:<br>JBEI-265990
K207-3-ompW-MatBC	E. coli K207-3 strain with MatBC genes integrated at ompW site	This work<br>JBEI registry:<br>JBEI-265991
BAP1 ΔbioH-RFP-RppA	BAP1-ΔbioH-RFP strain harboring the RppA-NT plasmid for flaviolin production	This work<br>JBEI registry:<br>JBEI-265992
K207-3-RppA	K207-3 strain harboring the RppA-NT plasmid for flaviolin production	This work<br>JBEI registry:<br>JBEI-265993
BAP1-ΔbioH-MatBC-RppA	BAP1-ΔbioH-MatBC strain harboring the RppA-NT plasmid for flaviolin production	This work<br>JBEI registry:<br>JBEI-265994
K207-3-ompW-MatBC-RppA	K207-3-ompW-MatBC strain harboring the RppA-NT plasmid for flaviolin production	This work<br>JBEI registry:<br>JBEI-265995

BAP1-ΔbioH-MatBC-mp6	BAP1-ΔbioH-MatBC strain harboring the mutagenesis plasmid mp6	This work JBEI registry: JBEI-265996
E1_S1	BAP1-ΔbioH-MatBC strain harboring the mutagenesis plasmid mp6 after 7 days of ALE – colony 11	This work JBEI registry: JBEI-265997
E1_S2	BAP1-ΔbioH-MatBC strain harboring the mutagenesis plasmid mp6 after 7 days of ALE - colony 12	This work JBEI registry: JBEI-265998
E1_S3	BAP1-ΔbioH-MatBC strain harboring the mutagenesis plasmid mp6 after 7 days of ALE - colony 13	This work JBEI registry: JBEI-265999
E1_S4	BAP1-ΔbioH-MatBC strain harboring the mutagenesis plasmid mp6 after 7 days of ALE - colony 15	This work JBEI registry: JBEI-266000
E1_S5	BAP1-ΔbioH-MatBC strain harboring the mutagenesis plasmid mp6 after 7 days of ALE - colony 16	This work JBEI registry: JBEI-266001
E1_S6	BAP1-ΔbioH-MatBC strain harboring the mutagenesis plasmid mp6 after 7 days of ALE - colony 18	This work JBEI registry: JBEI-266002
BAP1-ΔbioH-MatBC-Δbio-kan	E. coli BAP1 strain with MatBC genes integrated at bioH site and kanamycin resistance integrated at the biotin biosynthesis operon encoding the genes (*bioA, bioB, bioC, bioD, and bioF*)	This work JBEI registry: JBEI-266003
BAP1-ΔbioH-MatBC-Δbio-kan-mp6	BAP1-ΔbioH-MatBC-Δbio-kan strain harboring the mutagenesis plasmid mp6	This work JBEI registry: JBEI-266004
E2_S1	BAP1-ΔbioH-MatBC-Δbio-kan-mp6 strain harboring the mutagenesis plasmid mp6 during ALE - colony 1	This work JBEI registry: JBEI-266005
E2_S2	BAP1-ΔbioH-MatBC-Δbio-kan-mp6 strain harboring the mutagenesis plasmid mp6 during ALE - colony 2	This work JBEI registry: JBEI-266006
E2_S3	BAP1-ΔbioH-MatBC-Δbio-kan-mp6 strain harboring the mutagenesis plasmid mp6 during ALE - colony 3	This work JBEI registry: JBEI-266007
E2_S4	BAP1-ΔbioH-MatBC-Δbio-kan-mp6 strain harboring the mutagenesis plasmid mp6 during ALE - colony 4	This work JBEI registry: JBEI-266008
E2_S5	BAP1-ΔbioH-MatBC-Δbio-kan-mp6 strain harboring the mutagenesis plasmid mp6 during ALE - colony 5	This work JBEI registry: JBEI-266009
E2_S6	BAP1-ΔbioH-MatBC-Δbio-kan-mp6 strain harboring the mutagenesis plasmid mp6 duringALE - colony 6	This work JBEI registry: JBEI-266010

E2_S7	BAP1-ΔbioH-MatBC-Δbio-kan-mp6 strain harboring the mutagenesis plasmid mp6 duringALE - colony 7	This work JBEI registry: JBEI-266011
E2_S8	BAP1-ΔbioH-MatBC-Δbio-kan-mp6 strain harboring the mutagenesis plasmid mp6 during ALE - colony 8	This work JBEI registry: JBEI-266012
E2_S9	BAP1-ΔbioH-MatBC-Δbio-kan-mp6 strain harboring the mutagenesis plasmid mp6 during ALE - colony 9	This work JBEI registry: JBEI-266013
E2_S10	BAP1-ΔbioH-MatBC-Δbio-kan-mp6 strain harboring the mutagenesis plasmid mp6 during ALE - colony 10	This work JBEI registry: JBEI-266014
E2_S11	BAP1-ΔbioH-MatBC-Δbio-kan-mp6 strain harboring the mutagenesis plasmid mp6 during ALE - colony 11	This work JBEI registry: JBEI-266015
E2_S12	BAP1-ΔbioH-MatBC-Δbio-kan-mp6 strain harboring the mutagenesis plasmid mp6 during ALE - colony 12	This work JBEI registry: JBEI-266016
E2_S13	BAP1-ΔbioH-MatBC-Δbio-kan-mp6 strain harboring the mutagenesis plasmid mp6 during ALE - colony 13	This work JBEI registry: JBEI-266017
E2_S14	BAP1-ΔbioH-MatBC-Δbio-kan-mp6 strain harboring the mutagenesis plasmid mp6 during ALE - colony 14	This work JBEI registry: JBEI-266018
E2_S15	BAP1-ΔbioH-MatBC-Δbio-kan-mp6 strain harboring the mutagenesis plasmid mp6 after 2 weeks of ALE - colony 15	This work JBEI registry: JBEI-266019
E2_S16	BAP1-ΔbioH-MatBC-Δbio-kan-mp6 strain harboring the mutagenesis plasmid mp6 after 2 weeks of ALE - colony 16	This work JBEI registry: JBEI-266020
E2_S17	BAP1-ΔbioH-MatBC-Δbio-kan-mp6 strain harboring the mutagenesis plasmid mp6 after 2 weeks of ALE - colony 17	This work JBEI registry: JBEI-266021
BAP1-ΔbioH-MatBC-ΔfadI	BAP1-ΔbioH-MatBC strain with additional knockout mutation of fadI	This work JBEI registry: JBEI-266022
E1_S2 -RppA	E1_S2 evolved strain harboring the RppA-NT plasmid for flaviolin production	This work JBEI registry: JBEI-266023
E2_S3 -RppA	E2_S3 evolved strain harboring the RppA-NT plasmid for flaviolin production	This work JBEI registry: JBEI-266024
E2_S7 -RppA	E2_S7 evolved strain harboring the RppA-NT plasmid for flaviolin production	This work JBEI registry: JBEI-266025
E1_S17 -RppA	E2_S17 evolved strain harboring the RppA-NT plasmid for flaviolin production	This work JBEI registry: JBEI-266026

All strains used in this study are available through the Joint BioEnergy Institute's Inventory of Composable Elements (ICE), an open-source registry software and platform for managing information about biological parts (https://public-registry.jbei.org/folders/894).

Supplementary Table 2. Primers used in this study

Primer name	Sequence (5' to 3')
BioH integration F	5'gacatcatgcgctttaccgggctgtcagaagaagagttggctgcggcgag3'
BioH integration R	5'tcggcatagccagcataatcccggtactgttagcatatgttcatccttgt3'
OmpW integration F	5'cagcgtgaccaataacacgcaac3'
OmpW integration R	5'gcaagccgaatctatgcttgaatcc3'
Quick change R6kan backbone F	5'gtgcaccagatgcggtgtgaaataccgc3'
Quick change R6kan backbone R	5'cgcatctggtgcactctcagtacaatctgc3'
R6kan-bioH-MatBC-insert F	5'aagaaggagatatacatatggtgagcaaccatcttttcgacgc3'
R6kan-bioH-MatBC-insert R	5'cctggagatccttactcgagtcaaaccagcccgggcacga3'
R6kan-bioH-MatBC-BB F	5'tcgtgcccgggctggtttgactcgagtaaggatctccagg3'
R6kan-bioH-MatBC-BB R	5'tcgaaaagatggttgctcaccatatgtatatctccttcttaaattgttaa3'
R6kan-bioH-RFP-insert F	5'gcgagtcagtaaagttctgtctcgccatttcaaaagccaccgtaggctggagctgcttcg3'
R6kan-bioH-RFP-insert R	5'tatgttcatccttgtaagtcaaaagagaacaatagcggtataaacgcagaaaggcccacc3'
FadI-K/O F	5'agacgaacattaagggtaaacgctgatgtcatttccattattccgcctccagaaccattgccgcgccaagattccg gggatccgtcgacc3'
FadI-K/O R	5'atgatcaggtcagaccactttatttattttttacaggggagtgttccattgttagcggtttacgtacgcgtgtaggctg gagctgcttcg3'
R6kan-bioH-RFP-BB F	5'ctcttttgacttacaaggatgaacatatgctaacagtacatggctaattcccatgtcag 3'
R6kan-bioH-RFP-BB R	5'cgagacagaactttactgactcgccgcagccaacatctagatgtattcgcgagg 3'
R6kan-ompW*-MatBC-BB F	5'aagaaggagatatacatatggtgagcaaccatcttttcgacgc3'
R6kan-ompW*-MatBC-BB R	5'cctggagatccttactcgagtcaaaccagcccgggcacga3'
R6kan-ompw*-MatBC-BB R	5'tcgtgcccgggctggtttgactcgagtaaggatctccagg3'
R6kan-ompw*-MatBC-BB R	5'tcgaaaagatggttgctcaccatatgtatatctccttcttaaaagatct3'
FadLJI screen F	5'tcagcgcggattcatatagctttgacc3'
FadLJI screen R	5'cgtgtatgagcgcacaatatccaggg3'
FadAB screen F	5'ctgtagaccggataaggcgttcac3'
FadABscreen R	5'gatttctgccgagcgtgatcagat3'
BioH screen F	5' gggaacaatccaaagccctg 3'
BioH screen R	5' tgaacagaccgaacaaatccc 3'
OmpW screen F	5'gttctgcaaccgtacgtccaacag3'
OmpW screen R	5'gactggttacgtgacgcccacg3'
Bio-K/O F	5' ttactgatgagtttcatgaaccctcctttcttgtttgcagaaagtgtagccagaaaccctcacgcggacttctcgatt ccggggatccgt 3'

Bio-K/O R	5'ttagaatttaattggttaaattgcagtcaatcgaagacgcgatctcgctcgcaatttaaccaaatacagaatgggtgtaggctggagctg 3'

Supplementary Table 3. Genes used in this study

Gene name	Sequence (5' to 3')
matB	atggtgagcaaccatcttttcgacgccatgcgggccgccgcgcccggtaacgcaccattcatccggatcgataacacgcgcacatggacctatgacgacgccttcgctctttccggccgcattgccagcgcgatggacgcgctcggcattcgccccggcgaccgcgttgcggtgcaggtcgagaaaagtgccgaggcattgatcctctatctcgcctgtcttcgaagcggcgccgtctacctgccgctcaacaccgcctatacgctggctgagctcgattattttatcggcgatgcggagccgcgtttggtggttgtcgcatcgtcggctcgagcgggcgtggagacaatcgccaagccccgcggtgcgatcgtcgaaactctcgacgctgctggcagcggctcgttgctggatctcgcccgcgacgagccggccgactttgtcgatgcctcgcgctccgccgatgatctggcggcgatcctctacacgtccggaacgacgggacgctccaagggggcgatgctcacgcatgggaacctgctctcgaacgccctgaccttgcgagatttttggcgcgtcaccgccggcgatcgactgatccatgccttgccgatcttccacacgcatggactgttcgtcgccacgaacgtcacactgctcgccggcgcctcgatgttcctgctgtcgaagttcgacccggaggagatcctgtcgctgatgccgcaggcaacgatgctgatgggcgtgccgaccttctacgtgcgcctcctgcagagcccgcgcctcgacaagcaagcggtcgccaacatccgcctcttcatttccggttcggctccactgcttgcagaaacacataccgagttccaggcacgtaccggtcacgccattctcgagcgctacggcatgacggaaaccaatatgaacacgtccaacccttatgaggggaaacggattgccggaacggtcggcttcccgctgcctgatgtgacggtgcgcgtcaccgatcccgccaccgggctcgcgctgccgcccgaacaaaccggcatgatcgagatcaaggggccgaacgttttcaagggctattggcgcatgcccgaaaaaaccgcggccgaattcaccgccgacggtttcttcatcagcggcgatctcggcaagatcgaccgcgacggttatgtccacatcgtcggccgcggcaaggatctggtgatttcgggtggatacaacatctatccgaaagaggttgagggcgagatcgaccagatcgagggtgtggttgagagcgctgtgatcggcgtgccgcatcccgatttcggagaaggcgtaacggccgtcgtcgtgcgcaagcccggcgctgccctcgatgaaaaggccatcgtcagcgccctccaggaccggctcgcgcgctacaaacaacccaagcgcatcatctttgcagaggacttgccgcgcaacacgatgggtaaggttcagaaaaacatcctgcggcagcaatacgccgatctttataccaggacgtaa
matC	atgggtattgaattactgtccataggcctgctgatcgccatgttcatcattgcgacgatccagccaatcaacatgggtgcgctcgcctttgccggcgccttcgtgctcggctcgatgatcatcgggatgaaaaccaacgaaatatttgccggctttccgagtgatctgttcctgacgctcgtcgccgtcacctacctcttcgccatagcgcagatcaacggcacgatcgactggctcgtcgaatgtgccgtccgcctggtacgcgggcggatcggcttgattccctgggtgatgttccttgtcgccgccatcattactggcttcggtgcacttgggcctgctgcggtcgccattctcgcacccgtcgcgttgagctttgccgtgcagtaccgcattcatccggtgatgatgggtctgatggtgatccacggcgcgcaggcaggcggcttctcgccgatcagcatctatggcggaatcaccaaccagatcgttgcgaaggccggcctgcctttcgctccgacctcgctgtttctttccagcttcttctttaacctggcgatcgcggtgctggtgttcttcgtgttcggcggcgcgagggtgatgaagcacgatcccgcatcacttggccccttgcccgaactccatcccgagggcgtatcggcgtcgatcagaggccacggcggcacgccggcaaaaccgatcagagagcatgcctatggtacggcggccgataccgcgacgacgttgcgtctgaacaatgagagaattaccaccttgatcggcctgacggcgctcggcatcggcgccctggttttcaagttcaatgttggcctcgtcgccatgaccgtcgccgtcgtcctcgcgctgctgtcaccgaagacccagaaggccgcaatcgacaaggtcagttggtcgaccgtgctgctgattgccggcatcatcacctatgtcggcgtcatggagaaggccggtacggtcgactacgtggcgaatggcatatccagtctcggcatgccgctactggtagcgctcctgctttgctttacgggcgccatcgtctcggcctttgcttcctcgaccgcgctgctcggcgcgatcatcccgcttgccgttccattcctcctgcaagggcacatcagcgccatcggtgtggtcgcggcgatcgccatctcgacgacgatcgtcgacaccagcccattctccaccaacggcgcccttgtcgtcgccaatgcgccggacgacagccgtgagcaggtgttgcgacagctactgatctacagcgccttgatcgctatcatcggtccgatcgttgcctggttggtgttcgtcgtgcccgggctggtttga
mdcF	atgacatatgtaataattcacgcactagctccgatcttcgtgattatgctgctcggcttctgggcgggtaaagcgggcatggttgacaacaaaaacgtgagcctgctgaacatttttgtgatggattttgcactgccggcaaccctgttcagcgcgaccgttcagacgccgtgggctggtatcgttgcgcagagcccgctggtcctggtgctgaccggtgctatgtggattacgtacgcggcgatctactttctggctacgtctgtcttcaaacgcaccccgcaagatgccgcggttttgacgctgaccgttgcgttacctaattatgcggcgctgggcctgccgatcctgggcagcgttctgggtgagggtgccagcacctctttgtcggtggcggtgagcatcgcgtgcggttctgtcctaatgaccccattttgtctgctcattctggagcgcgaaaaagcccgtgcggcgggtgagaacagcggttccaccctggctatgcttccggttctgatgtggcgtagcgttaagaagccgattgtgtggggtccgttgcttggcgtggtgttatctgcgataggcatcaagatgccggacctgttattggcctcaattaagccgctgggcctggcagcgactgccgcggctctgttcttgactggtgtcatcctgagtgcacgtaaactgcagctgaatgcgctgattgccacctccaccattgtgaagctgctggtgcaaccgtttatcgcgtggggtttggttatgctgttgggcctccacggctccatcgctatcaccgcaattttgatgattgccttggctgcaggcttcttcggtgtagtttttggtaaccgttttggcgttcaaagcccggacgcggaagcagttttgctcctgtcgagcgtgctttgcatcctgtcccttccgctgttcatcagcttgaccagcggtctgtaa
madL	atggtaatatatggagttgctctattatcaggctgtttcctggtcggtaaggtgaccggttcctggttaggtgctgcgatcggcgtgaaagcgaacgttggcggcgttggcatctcgatgctgttgttggtcatcgtgtgcgacatcctgatcaagaagggtaagctgaagcaactgtctcaggacggcatcggcttttggaatgcaatgtacatcccgattgtagtggccatggcagcgaaacaaaacgtgattggtgcgattgatggtggttggcttgccctgttggcgggtggcgttgctaccgttgttagctatttcatgattccagttattagcaaactgggtcagggtacgaaagtgccggcgattgaagagccgagcagctaa
madM	atgtgggatatactagtagctcaattaacacagaacggtttaattacctccttcgtgattgtgggcgtgaccatgtatgttgcgtacatgctgtcagccaagttgaccaaaggtaaattccacggcagcgcgatcgcgatcattttgggcttggtcctggcgtacgtgggtggggccgcgactgacggcggttcgaaaggcctcgcgagcattccgatgtttgctggcgttggcctgatgggcggtagcatgctccgcgacctggccatcgtcgcgacctcgttcggtgtggatctgcgtgaggtgaagaagagcggtctgtccggcaccgtttctctgttcatgggtatttttgttagcttcttcatcggtgctggtatggcaatcgcgtttggttataccgatgcagtgagcatcactaccattggtgcgggcgcggtgacgtacattgttggtccggttaccggtgcggcggtcggagcggatagcgcggtagttgcgctgtctgttgctgctggcttggttaagtccatcctggtgatgacctccaccccgctgttcgccaagatcgtgggactggacaacccgaacagcgcaatggtctttggtggcttaatgggtacgacctctggcgtgaccgcaggcttggcagcaactaatccaaaattggttccgtacggtgctatgacgagcaccttttatacgggtctgggttgtctgctttgcccgagcgttctgtttctgctgatgaaaggcatctacggctaa
rfp	atggcgagtagcgaagacgttatcaaagagttcatgcgtttcaaagttcgtatggaaggttccgttaacggtcacgagttcgaaatcgaaggtgaaggtgaaggtcgtccgtacgaaggtacccagaccgctaaactgaaagttaccaaaggtggtccgctgccgttcgcttgggacatcctgtccccgcagttccagtacggttccaaagcttacgttaaacacccggctgacatcccggactacctgaaactgtccttcccggaaggtttcaaatgggaacgtgttatgaacttcgaagacggtggtgttgttaccgttacccaggactcctccctgcaagacggtgagttcatctacaaagttaaactgcgtggtaccaacttcccgtccgacggtccggttatgcagaaaaaaaccatgggttgggaagcttccaccgaacgtatgtacccggaagacggtgctctgaaaggtgaaatcaaaatgcgtctgaaactgaaagacggtggtcactacgacgctgaagttaaaaccacctacatggctaaaaaaccggttcagctgccgggtgcttacaaaaccgacatcaaactggacatcacctcccacaacgaagactacaccatcgttgaacagtacgaacgtgctgaaggtcgtcactccaccggtgcttaa

Supplementary Figures

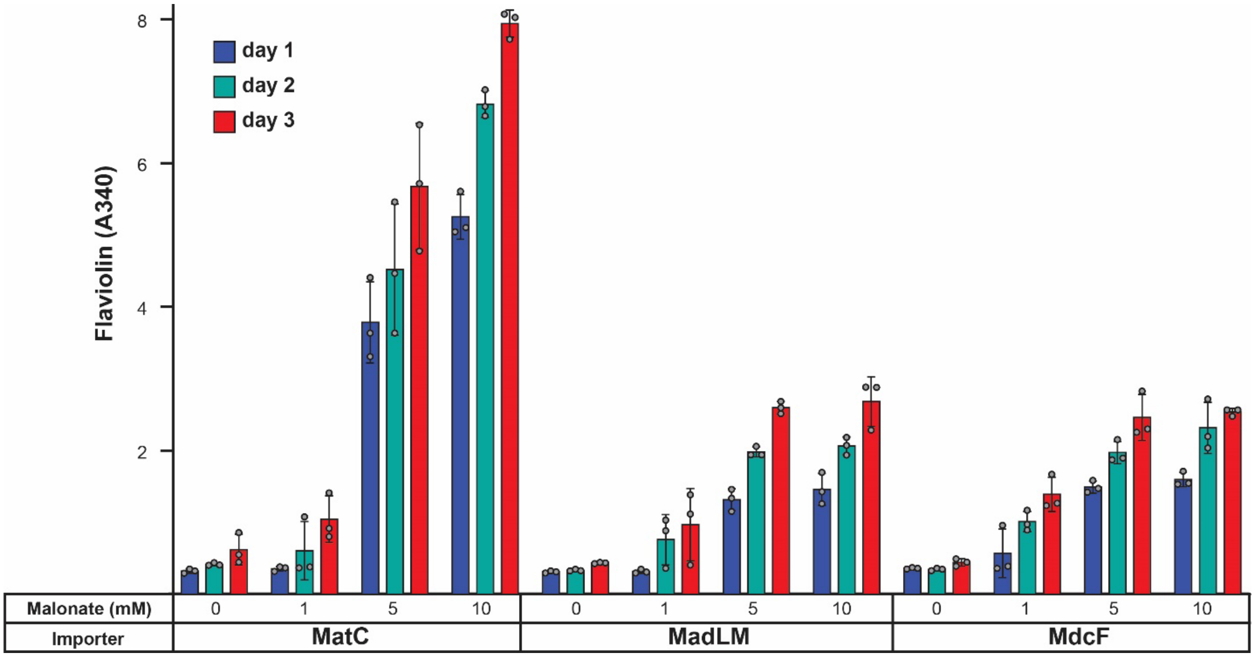

Supplementary Figure 1: **Screening plasmids containing the malonate importers MatC, MadLM, and MdcF along with the malonyl-CoA ligase MatB for increased production of flaviolin in the presence of malonate.** A340 absorbances, corrected for a pathlength of 1 cm, were recorded after 1, 2, or 3 days for three biological replicates of *E. coli* BAP1 strains harboring malonate importer plasmids (pCKmatBC, pBbA2c-MatB-MadLM, and pBbA2c-MatB-MdcF) along with the flaviolin producing RppA-NT plasmid. The strains were grown in LB medium with 2% glucose, 0.2% arabinose, 10 µM IPTG, and increasing concentrations of malonate. Plasmids bearing *madLM* and *mdcF* proved toxic when induced with 10 nM anhydrotetracycline, however, their leaky expression (no addition of inducer) could be tolerated. Improved flaviolin titers were observed in accordance with increasing malonate for all importer systems. All data are presented as mean values ± standard deviation (SD).

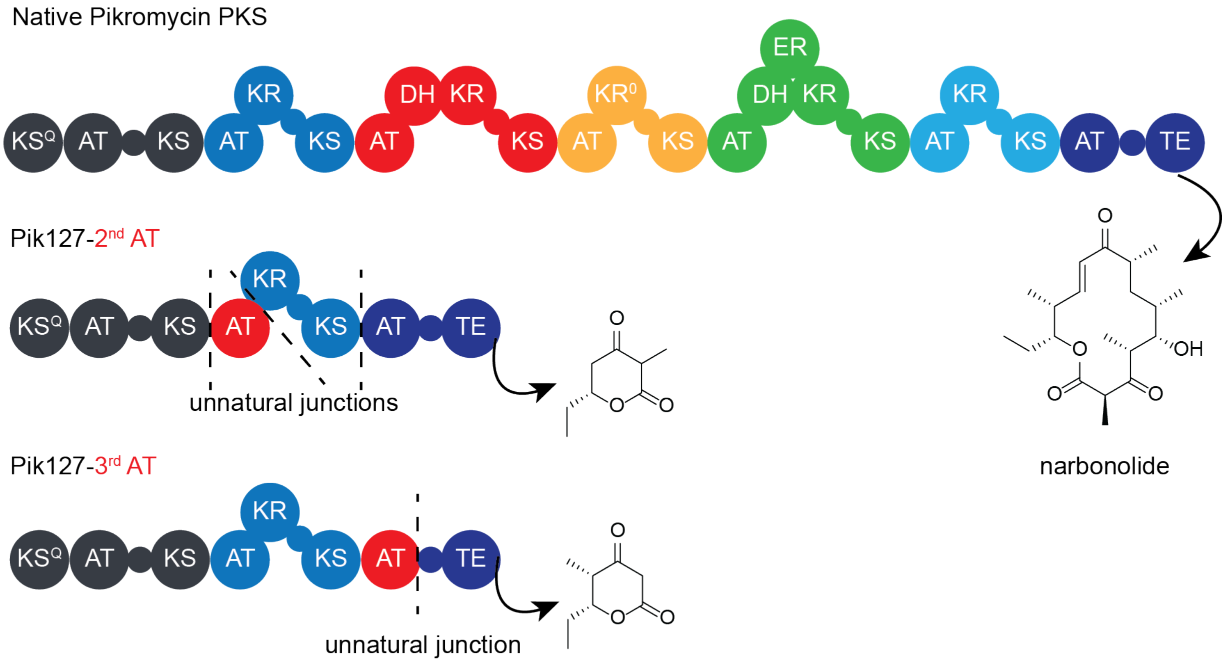

Supplementary Figure 2: **Structure of the native pikromycin biosynthetic gene cluster (BSGC) and hybrid pikromycin constructs.** (Top) Pikromycin native BSGC and domain structure and corresponding natural product narbonolide. (Bottom) Hybrid pik127 derivatives with the pikromycin module 3 AT (red) in the first (Pik127-2nd AT) and second (Pik127-3rd AT) extension modules. Introduction of pikromycin module 3 AT in the first module (Pik127-2nd AT) results in a hybrid construct with three unnatural junctions, while the Pik127-3rd AT construct only has one unnatural junction.

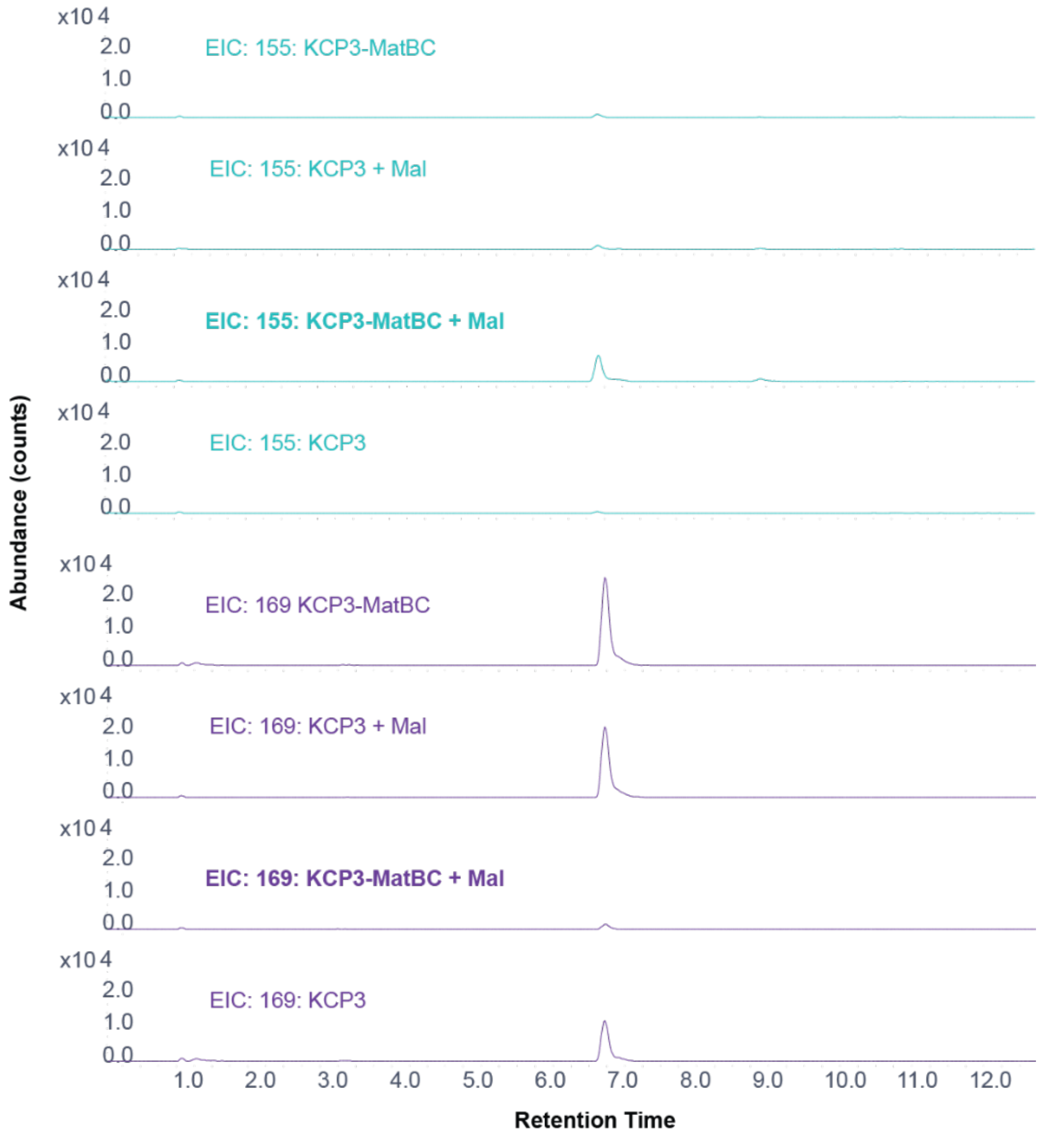

Supplementary Figure 3: **Representative LCMS traces of products produced by the pik127 strains KCP3 and KCP3-MatBC when supplemented with or without malonate**. The product 6-ethyl-5-methyldihydro-2H-pyran-2,4(3H)-dione elutes at 6.91 min with an m/z = 155 Da. The product 6-ethyl-3,5-dimethyldihydro-2H-pyran-2,4(3H)-dione elutes at 7.0 min with an m/z = 169 Da.

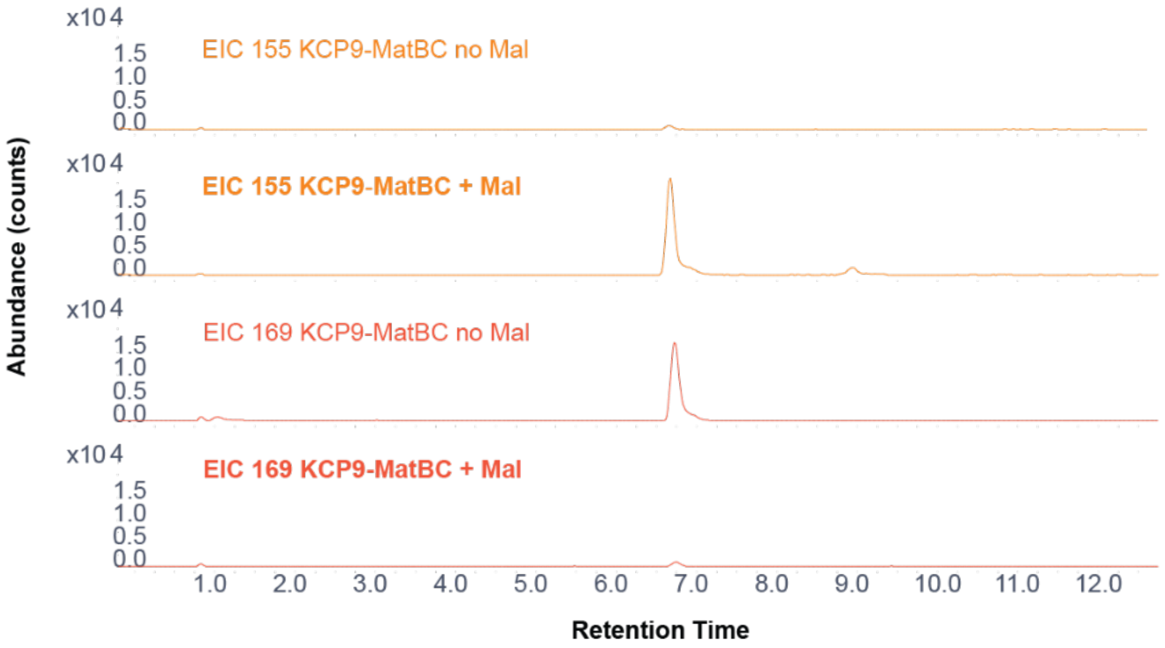

Supplementary Figure 4: **Representative LCMS traces of products produced by the pik127 strain KCP9-MatBC when supplemented with or without malonate.** For KCP9, cyclized products were only detectable in KCP9-MatBC cultures. No product was observed in the strain without MatBC (KCP9). While the standard for the KCP9 product 6-ethyl-3-methyldihydro-2H-pyran-2,4(3H)-dione could not be obtained, we observed that the KDP9 product had a similar retention time and pattern to that of the KCP3 strains of increased titer and ratiometric excess in the presence of malonate was observed. This leads us to believe that the peak observed with a m/z of 155 Da with a retention time of 6.91 min is the expected compound 6-ethyl-3-methyldihydro-2H-pyran-2,4(3H)-dione. The product 6-ethyl-3,5-dimethyldihydro-2H-pyran-2,4(3H)-dione elutes at 7.0 min with an m/z = 169 Da.

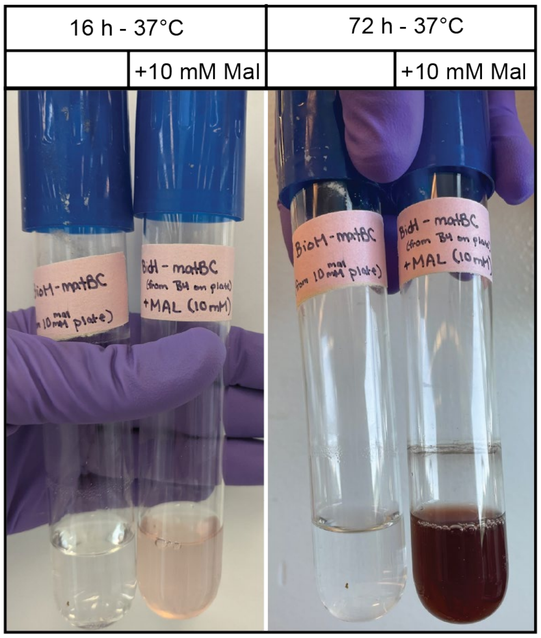

Supplementary Figure 5: **Flaviolin production in M9 minimal media.** BAP1-ΔBioH-MatBC strains carrying the RPPA-NT plasmid were cultured in M9 minimal medium with 2% glucose, 0.2% L-arabinose, and ±10 mM malonate. In malonate-supplemented conditions, cultures appeared light pink after 16 hours and gradually turned dark red and opaque by 72 hours at 37°C. In contrast, no color change or opaqueness was observed in the control lacking malonate supplementation.

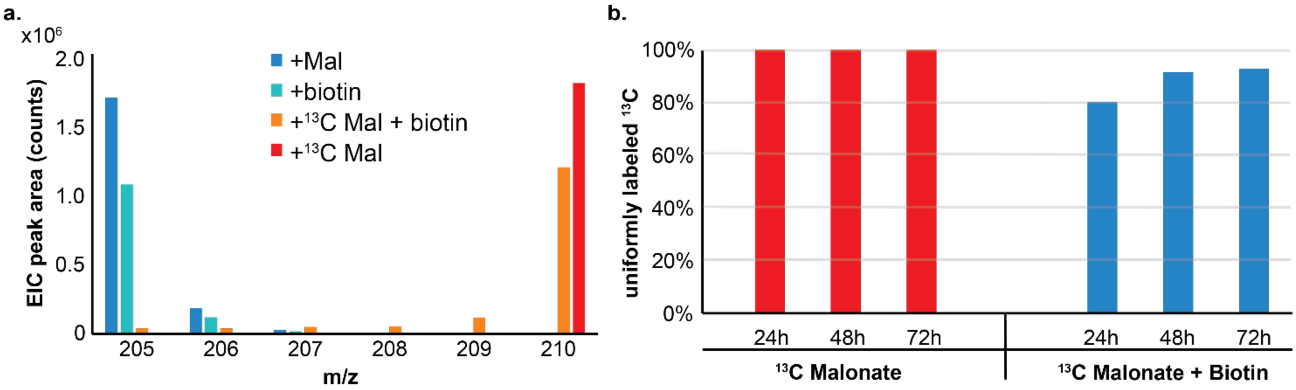

Supplementary Figure 6: **Isotopic abundance of ^{13}C flaviolin when produced in various media compositions.** (a) Integration of peak areas from the extracted ion chromatogram (EIC) of LC-MS traces run in negative mode. Peak areas represent the isotopic distribution of flaviolin under different conditions: malonate, biotin, [^{13}C]malonate + biotin, and [^{13}C]malonate. Supplementation with malonate or biotin resulted in major peaks corresponding to the natural isotopic abundance of flaviolin (m/z = 205), while [^{13}C]malonate led to peaks indicative of fully labeled flaviolin with five ^{13}C atoms (m/z = 210). When both [^{13}C]malonate and biotin were added, flaviolin species labeled with zero to five ^{13}C atoms were observed, with the majority carrying five ^{13}C carbons.(b) Time-course analysis of uniformly labeled (five ^{13}C carbons) flaviolin production relative to flaviolin labeled with zero to four ^{13}C carbons. Under [^{13}C]malonate supplementation, nearly 100% uniform labeling was maintained across all time points. When both [^{13}C]malonate and biotin were supplied, the proportion of fully labeled flaviolin increased from 79.9% at 24 hours to 92.6% by 72 hours. Final absorbance measurements at 72 hours were recorded at 340 nm and 600 nm in a 1 cm light path: [^{13}C]malonate: OD600 = 5.5, A340 = 14.78, A340/OD600 = 2.7, [^{13}C]malonate + biotin: OD600 = 5.1, A340 = 13.65, A340/OD600 = 2.7

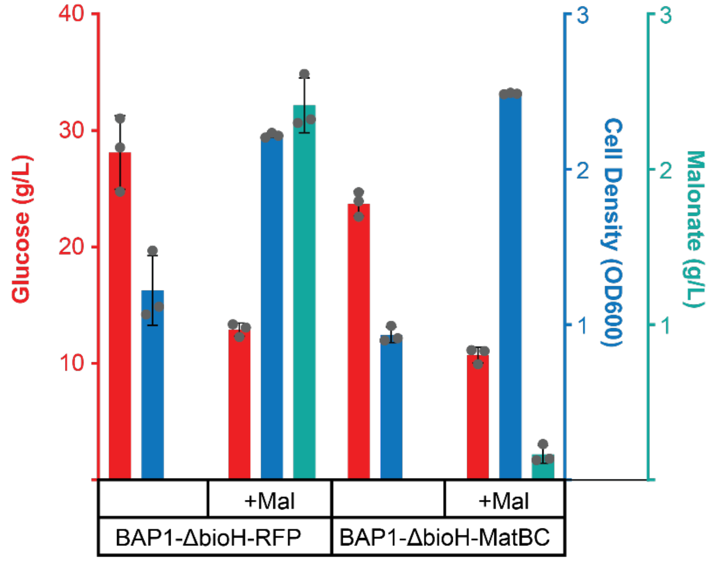

Supplementary Figure 7: **Monitoring malonate consumption in engineered strains.** Residual glucose and malonate in supernatants when three biological replicates of the strains BAP1-ΔbioH-RFP and BAP1-ΔbioH-MatBC were cultured in M9 minimal medium with 2 % glucose, 10 µM IPTG, 25 mg/L biotin, and +/- 20 mM malonate at 37°C for 24 h. Residual concentrations do not take into account the effects of evaporation. In both strains, when supplemented with malonate, the OD600 significantly increased, indicating that supplemental malonate in these conditions has a positive effect on cell growth. However, the concentrations of residual malonate were significantly depleted in the MatBC strain vs. the RFP control, indicating increased uptake of malonate in the MatBC strain to near depletion. All data are presented as mean values ± standard deviation (SD).

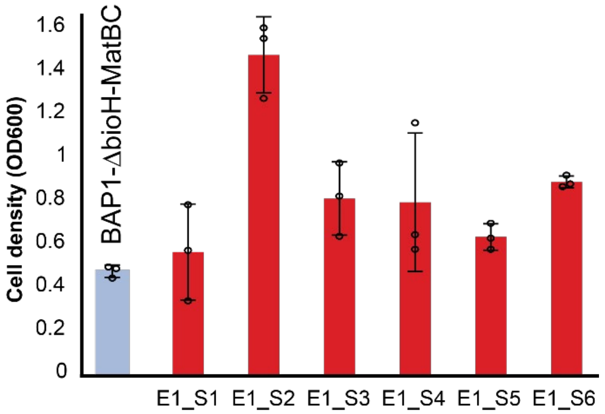

Supplementary Figure 8: **Cell growth after first round of adaptive laboratory evolution of BAP1-ΔbioH-MatBC strain.** OD_{600} of three biological replicates of the evolved strains cultivated in M9 minimal medium supplemented with 2 % glucose and 1 mM malonate in 24-well plates for 24 h at 37 °C. Stains E1_S2, E1_S5, E1_S3, and E1_S6 grew to significantly higher (p-values range from <0.0001-0.0277) cell density compared to BAP1-ΔbioH-MatBC. All data are presented as mean values ± standard deviation (SD). Statistical comparisons between groups were performed using GraphPad data analysis software with an unpaired t-test, assuming a Gaussian distribution, to calculate p-values and determine statistical significance.

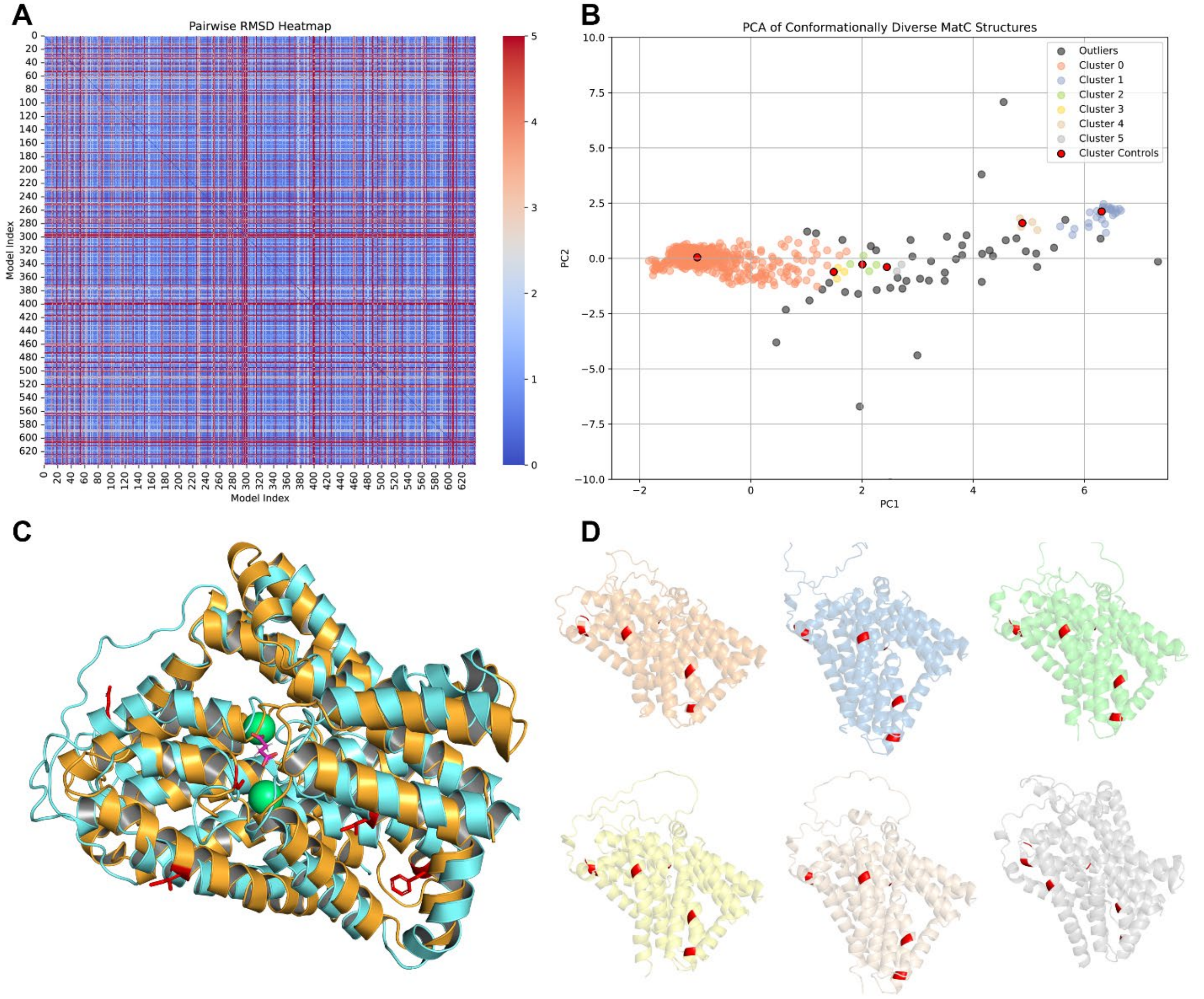

Supplementary Figure 9: **Predicting Conformations of MatC.** (A) Pairwise RMSD heatmap of all 640 structures with structure indices labeled on x and y axes. Colormap corresponds to RMSD in angstroms (colors capped at 5Å). (B) Principal component analysis (PCA) plot with clustering and cluster controls. (C) Structural alignment of PCA cluster 0 control conformation (orange) with VcINDY-NA+ in complex with succinate (cyan) (PDB: 6OL1) (RMSD = 4.46Å). (D) Predicted conformation space of MatC from PCA clustering. Observed residue mutations highlighted in red. (orange = cluster 0, blue = cluster 1, green = cluster 2, yellow = cluster 3, brown = cluster 4, grey = cluster 5).

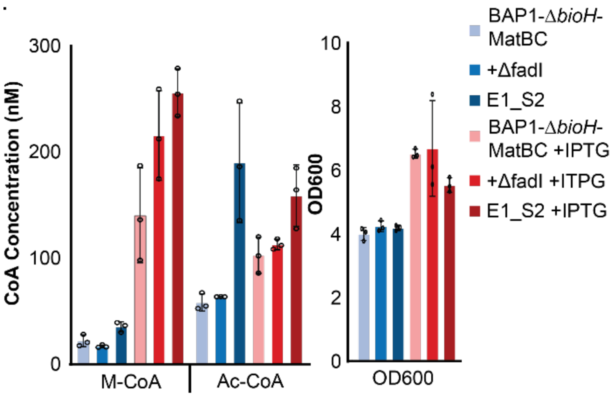

Supplementary Figure 10: **Analysis of reengineered ΔfadI strain.** LC-MS analysis of M-CoA and Ac-CoA concentrations of three biological replicates of the BAP1-ΔbioH-MatBC, BAP1-ΔbioH-MatBC-ΔfadI (+ΔfadI), and E1_S2 evolved strain when cultured in M9 minimal medium with 50 mM malonate and ± 10 µM IPTG. In all conditions, the evolved strain had a significantly increased M-CoA (no IPTG $p = 0.0354$, + IPTG = 0.0162) and Ac-CoA (no IPTG $p = 0.0160$, + IPTG = 0.0442) concentrations compared to BAP1-ΔbioH-MatBC. In all conditions, the ΔfadI strain possessed no statistically significant difference in M-CoA concentration compared to either BAP1-ΔbioH-MatBC or E1_S2. Statistical comparisons between groups were performed using GraphPad data analysis software with an unpaired t-test, assuming a Gaussian distribution, to calculate p-values and determine statistical significance.

Supplementary References

1. Jeschek, M. *et al.* Biotin-independent strains of Escherichia coli for enhanced streptavidin production. *Metab. Eng.* **40**, 33–40 (2017).
2. Lee, T. S. *et al.* BglBrick vectors and datasheets: A synthetic biology platform for gene expression. *J. Biol. Eng.* **5**, (2011).
3. Incha, M. R. *et al.* Leveraging host metabolism for bisdemethoxycurcumin production in Pseudomonas putida. *Metab Eng Commun* **10**, e00119 (2020).
4. Badran, A. H. & Liu, D. R. Development of potent in vivo mutagenesis plasmids with broad mutational spectra. *Nat. Commun.* **6**, 8425 (2015).
5. Wang, Z. *et al.* A microbial platform for recyclable plastics with customizable properties. *Research Square* (2023) doi:10.21203/rs.3.rs-3171588/v1.
6. Datsenko, K. A. & Wanner, B. L. One-step inactivation of chromosomal genes in *Escherichia coli* K-12 using PCR products. *Proc. Natl. Acad. Sci. U. S. A.* **97**, 6640–6645 (2000).
7. Pfeifer, B. A., Admiraal, S. J., Gramajo, H., Cane, D. E. & Khosla, C. Biosynthesis of complex polyketides in a metabolically engineered strain of E. coli. *Science* **291**, 1790–1792 (2001).
8. Murli, S., Kennedy, J., Dayem, L. C., Carney, J. R. & Kealey, J. T. Metabolic engineering of Escherichia coli for improved 6-deoxyerythronolide B production. *J. Ind. Microbiol. Biotechnol.* **30**, 500–509 (2003).
